# Searching for Amaranthin—A Multipotential Betacyanin from Natural Sources and In Vitro Cultures

**DOI:** 10.3390/ijms27125393

**Published:** 2026-06-15

**Authors:** Małgorzata Jeziorek

**Affiliations:** Department of Pharmaceutical Biology, Faculty of Pharmacy, Medical University of Warsaw, 02097 Warsaw, Poland; mjeziorek@wum.edu.pl

**Keywords:** amaranthin, betacyanin, betalain, plant in vitro culture, plant biotechnology, callus culture, suspension culture, natural pigments

## Abstract

Amaranthin is a major red-violet betacyanin of *Amaranthaceae* and an increasingly relevant natural pigment for food, cosmetic, nutraceutical, and biotechnological applications. This review integrates knowledge from over 100 studies, addressing amaranthin as a chemically defined betalain, distinguishing it from other scientific uses of the term, and evaluates its natural sources, analytical methods, extraction strategies, in vitro production systems, biosynthetic regulation, and biological activity. Cultivated *Amaranthus* species are among the richest plant sources, with total betacyanins of 46.1–199 mg/100 g fresh weight and amaranthin comprising, on average, 80.9% of the pigment fraction. Reliable identification and quantification rely on high-performance liquid chromatography coupled with a diode array detector (HPLC-DAD), liquid chromatography-tandem mass spectrometry (LC-MS/MS), and ultraviolet–visible (UV–Vis) spectrophotometry. Microwave- and ultrasound-assisted extraction can improve pigment recovery under optimized conditions, although its stability depends strongly on pH, temperature, solvent, time and storage parameters. While plant in vitro cultures, including callus, suspension, and shoot systems, have clarified biosynthetic regulation and offer controlled production platforms, engineered yeast systems have recently expanded production options, with *Yarrowia lipolytica* reaching 2.97 ± 0.029 g L^−1^ amaranthin in fed-batch fermentation. Amaranthin-rich extracts and amaranthin-type pigments show antioxidantand anti-inflammatory potential, while antimicrobial and antiviral activities have mainly been reported for mixed betacyanin fractions; direct mechanistic, bioavailability, and in vivo evidence for purified amaranthin remains limited. Standardized analytical protocols, further investigation of stable high-yield sources, physicochemical stability assessment, and structure–activity studies are identified as priorities for advancing future application-oriented research on this multipotential pigment.

## 1. Introduction

Amaranthin, the predominant betacyanin pigment in the *Amaranthus* genus, is a natural red-violet colorant with promising commercial applications across the food, pharmaceutical, and cosmetic industries [1,2,3,4]. Betacyanins, a subclass of betalains, are water-soluble nitrogenous pigments responsible for the characteristic coloration in *Amaranthaceae* species, which have been cultivated for centuries for their ornamental, nutritional, and medicinal properties [5,6,7,8].

Research on amaranthin content in both wild and cultivated *Amaranthaceae* species, with a simultaneous focus on plant in vitro systems, has emerged as a critical area of inquiry due to the increasing global demand for natural food colorants and antioxidants [1,9,10,11]. The practical significance of this research lies in the potential of amaranth-derived betacyanins as natural colorants with antioxidant properties, offering viable alternatives to synthetic dyes while contributing to innovation in the food industry [1,2,12,13]. The increasing consumer demand for natural additives, coupled with the estimated growth of the natural colorant market, underscores the importance of exploring amaranthin-rich plant tissues, as they may offer advantages over both synthetic dyes and other natural pigments like anthocyanins [1,14,15,16]. Global market trends consistently indicate a growing preference for natural pigments over synthetic alternatives, further emphasizing the importance of developing efficient extraction and analytical methods for amaranthin-like compounds [1,13,17,18].

Over the past two decades, significant advances in chromatographic and mass spectrometric techniques have enabled detailed profiling of betalain compounds, highlighting the predominance of amaranthin and isoamaranthin in cultivated amaranths [8,19,20,21]. High-resolution liquid chromatography–tandem mass spectrometry (LC-MS/MS), together with complementary spectroscopic and chromatographic approaches has substantially improved betalain identification and quantification, providing comprehensive compound catalogs and analytical protocols for profiling amaranthin and related betalains in diverse *Amaranthus* species [19,20,21,22].

Recent advances in genomics and analytical techniques have expanded our understanding of betalain pigment biosynthesis and regulation, revealing the molecular mechanisms underlying their production and accumulation [23,24,25,26]. Genomic resources and functional studies have identified key pathway enzymes, regulatory genes, and tissue-specific control mechanisms in *Amaranthus*, with chromosome-scale genome assemblies enabling the isoform-resolved annotation and mapping of betalain regulation across different tissues [23,24,25]. These discoveries have elucidated the core betalain biosynthesis pathway and identified transcription factors involved in betalain metabolism, further highlighting the significance of these pigments in food, pharmaceutical, and cosmetic applications [23,24,25,26].

Beyond summarizing the available literature, the specific objective of this review is to integrate these fragmented data into a coherent framework that highlights conceptual and technological bottlenecks in amaranthin research and production. The intention is to distill from this synthesis both an updated view of amaranthin as a multipotential pigment and a set of concrete, experimentally tractable questions that can guide future basic and applied research.

## 2. Literature Search Strategy and Thematic Organization

Because this article is a narrative and integrative review rather than a formal systematic review, the present section briefly describes the literature search strategy and the criteria used to identify and thematically organize publications dealing specifically with amaranthin as a betacyanin pigment.

To comprehensively investigate the research devoted to amaranthin as a betacyanin pigment, a multi-stage literature search was conducted, utilizing primarily the Scopus and Web of Science databases. The goal was to identify and compile publications detailing the plant sources of amaranthin as a betacyanin pigment, with particular emphasis on the plant in vitro culture techniques used to obtain this compound.

The initial literature search involved the keywords: “amaranthin”, “amaranthine”, “amarantin”, and “amarantine”. These were applied to the title, abstract, and keyword fields in the above-mentioned databases, resulting, in both cases, in over 200 publications. This preliminary search revealed that terms connected with amaranthin are used variably across different disciplines, indicating the need for a more refined search. A focused screening was then applied to capture literature specifically linking amaranthin to its identity as a betacyanin pigment by pairing the core terms with “betacyanin”, “betanin”, and “betalain” phrases. To identify experimental work involving controlled in vitro production systems, another search strategy was implemented using the core terms with additional keywords, such as “callus”, “suspension culture”, “hairy roots”, “transgenic roots”, “transformed roots”, “micropropagation”, and “bioreactor”. This collection of over 100 gathered and selected publications was further enriched with relevant records obtained by forward and backward citation chaining, supported by the AI-assisted SciSpace Deep Review tool, with final selection and verification performed by the author. Original experimental articles, reviews, or book chapters written in English and with an available full-text version were chosen for analysis, excluding those lacking essential experimental details. Full-text versions of publications were accessed through institutional subscriptions to platforms including ScienceDirect, PubMed/PubMed Central, and SpringerLink. Additional open-access articles were identified through a search of online resources and publisher repositories.

Finally, 136 of the most relevant publications and sources were selected and grouped into four thematic sections: (1) natural sources of amaranthin—documented occurrence of amaranthin in wild and cultivated species of the genus *Amaranthus* and other genera; (2) analytical and biosynthetic approaches—methods for the extraction, quantification, structural elucidation, and biosynthetic pathway analysis of amaranthin-type betacyanins; (3) plant in vitro systems—studies on amaranthin biosynthesis and production in cell, tissue, and organ cultures; and (4) biological activity—investigations of antioxidant, anti-inflammatory, and other bioactivities attributed to amaranthin as a betacyanin pigment. In addition, a brief section clarifies the different uses of the term “amaranthin” in scientific research that emerged from this extended literature survey.

## 3. Terminology Note: ‘Amaranthin’ in the Literature

Before focusing exclusively on amaranthin as a betacyanin, it is important to note that the term “amaranthin” has been used in the scientific literature to describe chemically and functionally distinct entities, including a plant betacyanin, a lectin, a seed storage protein, and a synthetic azo dye, which makes explicit clarification of the terminology essential. As the subject of this review, amaranthin refers specifically to the plant betacyanin pigment: betanidin 5-O-[β-D-glucuronosyl-(1→2)-β-D-glucoside] with full IUPAC name (2*S*)-5-[(2*S*,3*R*,4*S*,5*S*,6*R*)-3-[(2*R*,3*R*,4*S*,5*S*,6*S*)-6-carboxy-3,4,5-trihydroxyoxan-2-yl]oxy-4,5-dihydroxy-6-(hydroxymethyl)oxan-2-yl]oxy-1-[(2*E*)-2-[(2*S*)-2,6-dicarboxy-2,3-dihydro-1*H*-pyridin-4-ylidene]ethylidene]-6-hydroxy-2,3-dihydroindol-1-ium-2-carboxylate (PubChem CID: 6325284) [27]. However, across a broad spectrum of literature, the terms ‘amaranthin’, ‘amaranthine’, ‘amarantin’, and ‘amarantine’ appear with some limited spelling consistency in reported scientific papers. Therefore, being careful in this regard when reviewing the literature but taking into account all possible spellings ensures that some reports on the investigated topic are not overlooked.

In most cases, ‘amaranthin’ represents a vivid natural pigment of the betacyanin type [28], a lectin protein involved in plant defense mechanisms and biochemical studies [29,30], and a seed storage protein with applications in genetically engineered crops [31,32,33]. To supplement this data, it is worth mentioning here a synthetic red coloring known as Amaranth dye (E 123, Red Dye No.2, Food Red 9, Acid Red 27, Azorubin S, or Color Index (C.I.) number 16185), which is a modified red azo dye used as a food and beverage colorant and also in the cosmetics and textile industries [34]. Amaranth dye was banned as a food additive in the United States by the U.S. Food and Drug Administration (FDA) in 1976 as a suspected carcinogen but is still allowed in some countries for use, for example, in the European Union (EU) (as E 123), with quantity restrictions [35,36]. To complete this list, the term ‘amaranthine’ has also been metaphorically invoked in more abstract applications, retaining its adjectival sense of “everlasting”, “immortal”, or “imperishable”; examples include “amaranthine adventure” and “amaranthine scale” in biology [37,38], as well as its adoption as the name of a 3D-printed humanoid robot in robotics [39]. In Table 1, examples of various amaranthin contexts in scientific publications are listed.

The following sections of this review are devoted exclusively to amaranthin as a betacyanin pigment, using the nomenclature and structure details as presented by the NCBI PubChem databases [27].

## 4. Amaranthin Characteristics and Its Natural Sources

Research on the methods and techniques of amaranthin betacyanin separation, identification, and quantification has emerged as a critical area of inquiry due to the increasing demand for natural food colorants and bioactive compounds with health benefits. Betalains, including betacyanins and betaxanthins, are nitrogen-containing pigments responsible for the red-violet and yellow-orange colors in plants of the Caryophyllales order, such as *Amaranthus* species [19,43]. Since the early isolation of betanin from red beetroot in 1918, research has expanded to characterize betalains in various *Amaranthaceae* species, recognizing amaranthin as the predominant betacyanin in the *Amaranthus* genus [1,19] and also present in other genera of this family, for example: *Chenopodium*, *Celosia*, *Atriplex*, *Alternanthera*, *Gomphrena*, and *Iresine* [44,45,46,47,48,49,50].

### 4.1. Structure Characteristics and Analytical Methodology

**Figure 1:**
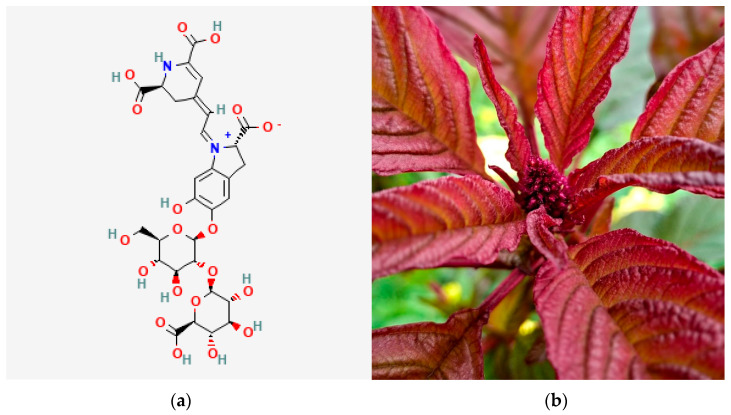
Characteristic amaranthin features: (**a**) amaranthin chemical structure [27]; (**b**) young *Amaranthus sp.* shoots presenting the typical color of the amaranthin presence (Photograph by D. Kucz).

Amaranthin is a disaccharide derivative that is a betanidin in which a beta-D-glucuronosyl-(1->2)-beta-D-glucosyl moiety is attached at position 5 (Figure 1a) [27]. It presents a characteristic amaranth color belonging to the betacyanin group of compounds found in *Amaranthus* sp., among others (Figure 1b).

The C-15 epimer of amaranthin–isoamaranthin is also found to be an accompanying compound in investigated plant tissues [2,11,51]. As for separation, identification, and quantification techniques, high-performance liquid chromatography (HPLC), LC-MS/MS, and high-speed counter-current chromatography (HSCCC) are primary methods for separating and identifying amaranthin and related betacyanins. These techniques enable precise molecular formula determination and fragmentation pattern elucidation, which are essential for distinguishing structural isomers and diastereomers [19,21,52,53,54]. UV–Vis spectrophotometry (typical λmax ≈ 535 nm for amaranthin) is widely used for screening and routine quantification [43]. In complex matrices, it should be complemented by LC-MS/MS for definitive identification and isomer resolution. Comparative studies indicate that HPLC methods are preferred when interfering substances are present, as spectrophotometric methods may show discrepancies of up to 15% with degraded samples [55].

Matrix-assisted laser desorption/ionization quadrupole ion trap time-of-flight mass spectrometry (MALDI-QIT-TOF MS) has also been applied for rapid identification without prior purification [52]. Recently, an attenuated total reflectance–Fourier transform infrared (ATR–FTIR) spectroscopy method was proposed by López et al. (2023) as a simple and efficient protocol for amaranth betalain extraction and stability analysis [20]. Recent studies emphasize tandem MS and NMR for confident structure confirmation [53,56]. Structural characterization among betacyanins and, more broadly, betalains is based on amaranthin’s characteristic unique glucuronosylglucosyl moiety and acylation patterns, which distinguish it from the well-known betacyanin betanin (beetroot red; E 162). Comparative evaluations of analytical methods highlight the complementarity of chromatographic and spectrometric approaches for amaranthin analysis. HPLC coupled with diode array and mass spectrometric detection (DAD/MS) enables accurate quantification and identification [19,47,57]. The integration of spectral data and fragmentation patterns enhances reliability in complex plant matrices [52,58]. HSCCC is recognized as complementary, especially for isolating diastereomers and enriching minor betacyanins [53,54]. Several studies demonstrate that structural variation within betalains modulates color-related properties, stability, and radical-scavenging capacity [45,59,60,61,62]. In particular, Cai et al. established a structure-antiradical activity series for purified *Amaranthaceae* betalains, revealing that celosianins exhibit higher antiradical potency than amaranthin/isoamaranthin by 2,2-Diphenyl-1-picrylhydrazyl radical (DPPH) assay, with glycosylation of the aglycone and hydroxyl group number as key structural determinants [62]. For acylated amaranthin-type betacyanins, oxidation generated structurally distinct derivatives with modulated antiradical activity; importantly, such oxidized products retained significant cardioprotective potential in cardiomyoblasts [61]. When purified native pigments were directly compared, celosianin exhibited markedly higher antioxidant potency than amaranthin (IC_50_ 21.5 vs. 32.2 μg/mL, respectively) [45]. By contrast, studies on betalain-rich plant extracts, including those from amaranth, cactus pear, and beetroot, support their broad bioactivity and potential as natural colorants, but do not provide systematic structure-activity comparisons of individual pigments [4,63]. Taken together, the evidence indicates that structural diversity among betalains, including amaranthin-related pigments and their oxidation products, governs physicochemical and antiradical properties at the molecular level, while extract-based functional assays further support their relevance as natural food colorants and health-promoting agents [4,45,59,60,61,62,63,64].

### 4.2. Amaranthin in Wild and Cultivated Plant Species

Research demonstrates substantial variation in amaranthin content across the *Amaranthaceae* family. Table 2 presents a multi-exemplary list of research on plant species in which the presence of amaranthin was tested, and it is composed to present worldwide works on plants of wild and cultivated origin. The main goal of this summary is to present the range and the origin of the *Amaranthaceae* plants examined for the presence of amaranthin, to highlight the extraction methods used for plant material analysis, and to summarize the analytical methods applied to quantify this specific metabolite. However, works listed in Table 2 were not solely focused on the amaranthin content but also often concerned the determination of various betalains and other metabolite groups. Authors often also investigated the antioxidant properties or other biological activity potential of whole extracts. Variability in sample preparation, diversified extraction conditions, and differences in instrumentation sensitivity contribute to divergence across studies, which significantly limits cross-study comparability in amaranthin quantity among various species and plant material of diverse origin. Matrix complexity and pigment instability during handling introduce extra quantification challenges. Despite certain limitations in the direct comparison of results, all these works provide rich documentation of the dynamically developing topic focused on searching for sources of natural dyes, such as amaranthin, with a wide potential of applications.

Some of the research works listed in Table 2 are exceptionally significant. In a comprehensive study of 21 genotypes from seven *Amaranthus* species, conducted by Cai et al. (1998), total betacyanins ranged from 46.1 to 199 mg/100 g of fresh plant material (FW), with amaranthin comprising an average of 80.9% of the total betacyanins [7]. Cultivated species exhibited higher betacyanin concentrations than wild species and had substantially greater biomass [7]. A broader survey by Cai et al. (2001) [65], covering 37 species across eight genera in the *Amaranthaceae* family, revealed total betacyanin content ranging from 0.08 to 1.36 mg/g fresh weight [58]. Among amaranth accessions grown as leafy vegetables, total betacyanin concentrations varied markedly from 4.7 to 478.8 mg/100 g dry weight, with amaranthin and isoamaranthin identified as major constituents [1]. Specific amaranth cultivars demonstrated notable differences in betacyanin profiles. In *A. gangeticus* accessions, four betacyanin compounds were identified: amaranthin, isoamaranthin, betanin, and iso-betanin [2]. Similarly, *A. tricolor* genotypes contained the same four betacyanin compounds, with genotypes VA14 and VA16 showing particularly high amaranthin and isoamaranthin contents [11]. Weedy *Amaranthus* species also showed significant potential, with the *A. viridis* genotype WAV7 and *A. spinosus* genotype WAS13 demonstrating the highest concentrations of betacyanins among the accessions tested by Sarker and Oba (2019) [66].

**Table 2:** Natural sources of amaranthin: Documented occurrences of amaranthin in worldwide research studies—wild and cultivated plant species.

Plant Species	Origin of Plant Material	Part of Plant Investigated for Amaranthin	Amaranthin Presence	General Extraction Conditions	Analytical Techniques	Ref.
*Alternanthera* *brasiliana* (L.) Kuntze	Cultivated (greenhouse, Brazil)	Leaves; (dried)	Amaranthin dominant in 4.6 mg/g DW of total quantity of betacyanins	Extracted with milli-Q water in an ultra-turrax T25 homogenizer at room temperature (60 s)	HPLC-DAD-MS *	[47]
*Alternanthera* *brasiliana* (L.) Kuntze	Cultivated (greenhouse, Brazil)	Leaves, stems; (dried)	Amaranthin: 80.08 μg/g DW ** (leaves); 14.10 μg/g DW (stems)	Extracted with acetonitrile: H_2_O acidified with 0.1% formic acid (1:1, *v*/*v*); stirring in a vortex (60 s)	HPLC-DAD/ HPLC-ESI-QTOF-MS	[67]
*Alternanthera tenella* Colla	Cultivated (greenhouse, Brazil)	Leaves, stems (dried)	Amaranthin: 14.71 μg/g DW (leaves); 5.40 μg/g DW (stems)	Extracted with acetonitrile: H_2_O acidified with 0.1% formic acid (1:1, *v*/*v*); stirring in a vortex (60 s)	HPLC-DAD/ HPLC-ESI-QTOF-MS	[67]
*Alternanthera* *sessilis* (L.) DC. (two ecotypes: red leaves—RE, green leaves—GE)	Collected wild (South China)—then cultivated in a growth chamber	Leaves; (fresh)	Amaranthin in red leaves ecotype (RE) ~35× higher than in green leaves ecotype (GE)	Extracted with methanol (2 × 30 min), supernatant discarded; precipitation soaked in distilled H_2_O (30 min), centrifuged— supernatant investigated	UV–Vis spectrophotometry	[68]
*Alternanthera* *sessilis* (L.) DC.	Collected in field (Genting Sempah, Malaysia)	Whole aerial parts; (lyophilized)	Amaranthin: the highest 72.7 mg/g extract in ethyl acetate extract (50 °C, 24 h)	Samples extracted with various solvents (H_2_O, 100% EtOH, 70% EtOH, ethyl acetate, 80% MetOH), temp. and time, in an incubator shaker	UV–Vis spectrophotometry	[69]
*Alternanthera* *sessilis* (L.) DC.	Cultivated (field-local farm, Malaysia)	Leaves, stems; (lyophilized)	Amaranthin was the highest in ethyl acetate extract of leaves: 93.94 mg/g extract	Extracted independently with water, ethanol, ethyl acetate, or hexane	UV–Vis spectrophotometry	[70]
*Amaranthaceae* (37 species of 8 genera: *Achyranthes*, *Alternanthera*, *Amaranthus*, *Aerva*, *Celosia*, *Cyathula*, *Gomphrena*, *Iresine*)	40 genotypes tested of 37 species from 16 countries and regions; most of them grown in a greenhouse and a few planted in a field (Wuhan, China)	Seedlings, leaves, inflorescences, stems; (fresh, frozen to −18 °C)	Amaranthin—averaged 91.5% of total peak area for 0.08–1.36 mg/g FW * of total betacyanins	Extracted with 80% MeOH; purified	HPLC-DAD/ ESI-MS/ ESI-MS-MS	[58]
*Amaranthus dubius* Mart. ex Thell.	Cultivated (supermarket; Malaysia)	Red parts of leaves (fresh)	Amaranthin as 70% of total betacyanins	Extracted with a substrate: solvent ratio of 1:4 chilled 70% MeOH in a blender, followed by subfractionation	HPLC/ LC-MS/MS	[71]
*Amaranthus dubius* Mart. ex Thell.	Cultivated (local market, Selangor, Malaysia)	Leaves, tips (fresh)	Amaranthin the most abundant betacyanin; Total betacyanin content in subfractionated *A. dubius* reported as 121.5% higher than that without subfractionation	Extracted with a substrate: solvent ratio of 1:4 chilled 70% MeOH in a blender, followed by subfractionation	HPLC-PAD; LC-MS/MS	[72]
*Amaranthus* spp. (48 accessions incl. *A. cruentus* L., *A. tricolor* L., *A. hypochondriacus*)	Cultivated, wild and weedy (greenhouse USA)	All above-ground biomass (lyophilized)	Amaranthin dominant—mostly over 80% of betacyanin composition; wide range of total betacyanins: 4.7–478.8 mg/100 g DT *	Extracted with deionized water; optimized extraction time (20 min), temp. (30 °C)	HPLC-DAD/ LC-ESI-MS–MS	[1]
*Amaranthus* spp. (21 genotypes of 7 species incl. *A. cruentus* L., *A. caudatus* L., *A. tricolor* L., *A. hypochondriacus* L., *A. hybridus* L., *A. lividus* L., *A. paniculatus* L.*)*	Cultivated and wild (USDA, China collections)	Seedlings, leaves, inflorescences, branches and stem skin (fresh)	46–199 mg betacyanins/100 g FW; Amaranthin avg. 81% of total betacyanins	Plant material chilled, cut and blanched in 3–4 volumes of H_2_O (80 °C, 5 min), filtered; repeated until no color present; extracts cooled, centrifuged, supernatant dried under vacuum (40 °C)	HPLC-PDA, FTIR spectroscopy, UV–Vis spectrophotometry	[7]
*Amaranthus* spp. (5 varieties of 3 species: *A. cruentus* L., *A. hypochondriacus* L., *A. mantagazzianus*)	Cultivated (field, Argentina)	Leaves (freeze-dried)	Amaranthin: up to 448 µg/g DW in *A. hypochondriacus* FK 280-FH1	Plant material crushed and homogenized with a blender; 70% MeOH/H_2_O (*v*/*v*) and ammonium acetate added, shaken (30 min), filtered (0.45 µM);	LC/MS with DAD	[73]
*Amaranthus tricolor* L. *(cv. Valentina)*	Cultivated (field, Russia)	Leaves; (fresh)	Cold decreased amaranthin 1.5–3.5 fold; repeated stress increased amaranthin ~30% in young leaves	Extracted with H_2_O	Spectrophotometry	[74]
*Amaranthus tricolor* L.	Cultivated (Hong Kong, market)	Seedlings (freeze-dried)	Confirmed presence of amaranthin/isoamaranthin via MS peaks; rapid ID of betacyanins tested	Extracted in a vial with 80% MeOH (at 23 °C, 10 min, continuously vibrating vial), filtered (syringe filter, 0.2 μm) and immediately used for MALDI-QIT-TOF MS analysis	MALDI-QIT-TOF MS; HPLC-DAD	[52]
*Amaranthus tricolor* L. (4 genotypes)	Cultivated (field, Bangladesh; (plant collected at 30 days old) greenhouse, Japan)	Leaves (fresh)	Amaranthin most abundant betacyanin, the highest for VA14 genotype: 22.84 mg/100 g FW	Extracted with 80% MeOH (with 1% acetic acid); homogenized and placed in a shaker (15 h, 400 rpm.); purified	HPLC-UV–Vis/ LC-MS-ESI	[11]
*Amaranthus* *caudatus* L.	Cultivated (organic field, Portugal)	Flowers (lyophilized)	Amaranthin: 171 ± 1 mg/g extract, isoamaranthin: 38 ± 1 mg/g extract	Ultrasound-assisted extraction with H_2_O (5 g/L, 22 min, power of 500 W, 20 kHz frequency); extract filtered (syringe filter, 0.22 μm)	HPLC-DAD	[63]
*Amaranthus* *cruentus* L.	Cultivated (from seeds collected in Valladolid, Yucatan)	Leaves, inflorescences (lyophilized)	Amaranthin determined as one of six amaranthin type betalains present	Plant tissue powder ground with sorbent BondElut-C18 (room temp.); using SPE cartridge performed elution with H_2_O (0.1% acetic acid), then with MeOH:H_2_O (1:1, *v*/*v*; 0.1% acetic acid), eluents evaporated, resuspended in H_2_O (0.1% acetic acid)	UHPLC-UV/VIS-LTQ-Orbitrap Elite MS	[19]
*Atriplex hortensis* L. var. Rubra	Cultivated (botanical garden, Poland)	Leaves, stems, seeds (lyophilized)	Amaranthin and celosianin prominent; leaves: ~561 mg/100 g DE * in crude extract; higher in weak anion-exchange (WAX) purified extracts/fractions	Extracted (3×, room temp., 1 h) with 20% aqueous acetone acidified with 1% formic acid (*v:v*); centrifuged (2000× *g*, 10 min), supernatants combined, filtered (Büchner funnel, 5 cm of silica gel, reduced pressure), evaporated	DI-IM-MS; semi-prep HPLC; spectrophotometry	[45,75]
*Celosia argentea* L. *(C. argentea* var. *cristata* (L.) Kuntze (1), *C. argentea* var. *plumosa* (Burvenich) Voss (2)	Cultivated (1—experimental garden, Halle, Germany; 2—field, Wuhan, China)	Inflorescences (yellow, orange, red), leaves (red), epidermal layers of stem (red); (fresh)	Amaranthin/isoamaranthin: 480.8 nmol/g FW as highest in red leaves of *Celosia argentea* var. *cristata* (L.) Kuntze	Frozen in liquid N_2_, homogenized (mortar), extracted with 80% aq. MeOH (containing 50 mM ascorbate, 30 min), centrifugated (14,000 *g*, 10 min at 4 °C), betalain content quantified photometrically; for LC-MS, LC-ESI-TOF-MS amaranthin isolated by semi-prep. HPLC	Spectrophotometry; semi-prep HPLC; LC-MS; LC-ESI-TOF-MS	[46]
*Chenopodium* *quinoa* Willd. (selected varieties)	Grains from germplasm bank (National Agricultural University La Molina in Lima; from plants cultivated in Peru)	Grains	Amaranthin identified in 14 of 29 examined quinoa varieties: up to 144.1 mg/kg FW of BGQ-77 variety	Extracted in a 10 mM sodium acetate buffer supplemented with 10 mM sodium ascorbate	HPLC-PDA; ESI-MS; TOF/Q-TOF MS	[48]
*Gomphrena globosa* L.	Cultivated (local market, Poland)	Flowers (petals); (fresh)	Amaranthin separated among other betacyanins in three highly polar solvent systems by HSCCC	Macerated with 20% acetone (*v*/*v*), with EDTA and 10% ascorbic acid included—30 min, room temp.; after filtration eluates concentrated and freeze-dried	LC-DAD-ESI-MS/MS; HSCCC	[49]
*Iresine lindenii* Van Houtte	- (ornamental plant)	Leaves; (fresh)	Amaranthin identified among eighteen betacyanins/isobetacyanins	Extracted with H_2_O in a blender, filtrated; extract concentrated using freeze-drier; concentrated extract subjected to semipreparative HPLC for the isolation of betacyanins, including amaranthin	HPLC-DAD; LC-DAD-ESI-MS/MS; IP-HSCCC	[44]

Notes. * For definitions of abbreviations used in this table, please refer to the Abbreviations section at the end of the manuscript. ** FW/DW/DT/DE—fresh weight/dry weight/dry tissue/dry extract, respectively, as reported in the cited studies.

**Figure 2:**
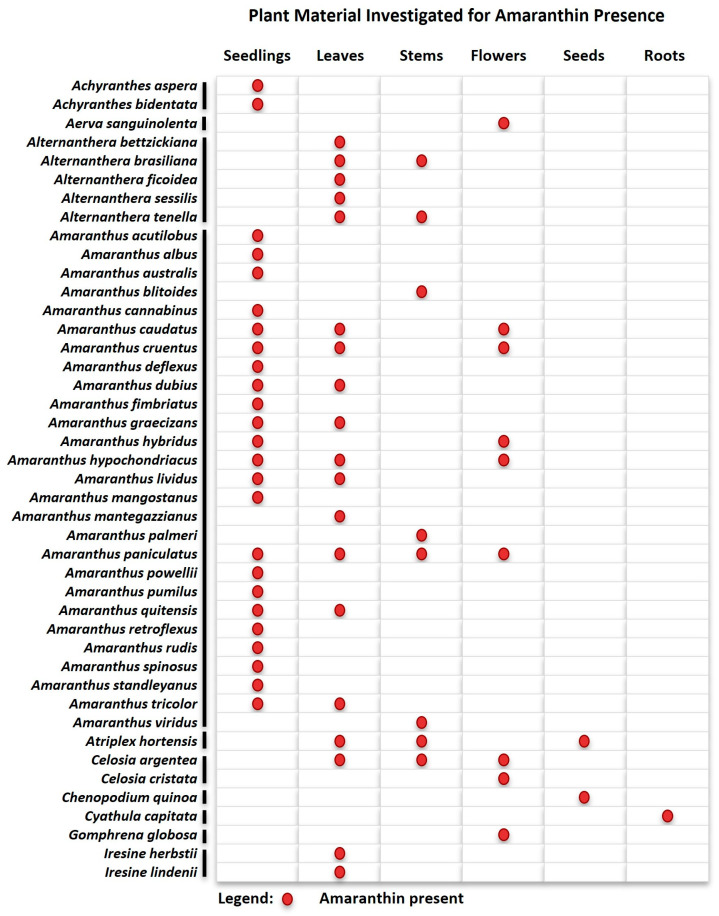
Plant species and plant materials investigated for amaranthin presence, including: seedlings, leaves, stems (incl. branches), flowers (incl. inflorescences), and seeds (incl. grains). The information supplements and extends the entries summarized in Table 2; all citations are as listed in Table 2.

To expand and facilitate the comparison between the taxa and tissues of the research presented in Table 2, the following Figure 2 shows the plant material from 43 species of 10 genera in which amaranthin has been investigated. There is a clear, significant predominance of research evidence supporting the presence of amaranthin in seedlings, primarily *Amaranthus* sp., and in leaves, here visibly expanding, for example, for *Alternanthera* sp.

### 4.3. Impact of Postharvest and Extraction Protocols on Amaranthin Yield and Stability

Postharvest and extraction protocols influence amaranthin stability during storage and processing, with temperature, pH, and antioxidants playing key roles [7,20,76]. Therefore, treatments such as freeze-drying, solar drying, and cold storage markedly affect betacyanin retention. Freeze-drying and low-temperature storage preserve pigment content better than air or oven drying [76,77]. Thermal and light exposure accelerate degradation, necessitating optimized handling protocols [59,76]. Visible/near-infrared spectroscopy can aid in the non-destructive monitoring of pigment stability [77]. Khandaker et al. (2009) examined seven cultivars of red amaranth (*Amaranthus tricolor* L.) [78]. They proved how variable factors such as the type of red amaranth cultivar, its degree of maturity, and food processing factors (temperature, light exposure, oxidant presence) quantitatively influenced betacyanin yields and preservation [78].

Conventional solvent extraction with water or ethanol is often optimized by multivariate analysis and remains widely used for amaranthin recovery [15,76]. Emerging methods such as microwave-assisted and ultrasound-assisted extraction can enhance pigment yield and purity while preserving its stability [63,79,80]. Applying spectroscopic methods, such as attenuated total reflectance–Fourier transform infrared (ATR-FTIR) spectroscopy, assists in monitoring pigment stability post-extraction [20]. These gradually implemented methods represent advances toward greener, more efficient pigment recovery and quality evaluation, which are crucial for amaranthin application.

Despite advances in betalain research, challenges still remain in the reliable separation, identification, and quantification of amaranthin due to its chemical diversity and instability under processing conditions [19,57]. Discrepancies in extraction protocols, solvent systems, and analytical parameters persist [12,52,53]. Moreover, the structural complexity of amaranthin, characterized by glucuronosylglucoside moieties and acylation patterns, complicates its differentiation from other betacyanins and betalains [53,77]. Discussion regarding the optimal methods for preserving pigment stability during extraction and storage, which impacts quantification accuracy and application potential, is also present [20,76]. The lack of standardized, high-throughput approaches limits the comprehensive understanding and commercial exploitation of amaranthin-rich extracts [1,15].

## 5. Plant In Vitro Systems for Amaranthin Biosynthesis

**Figure 3:**
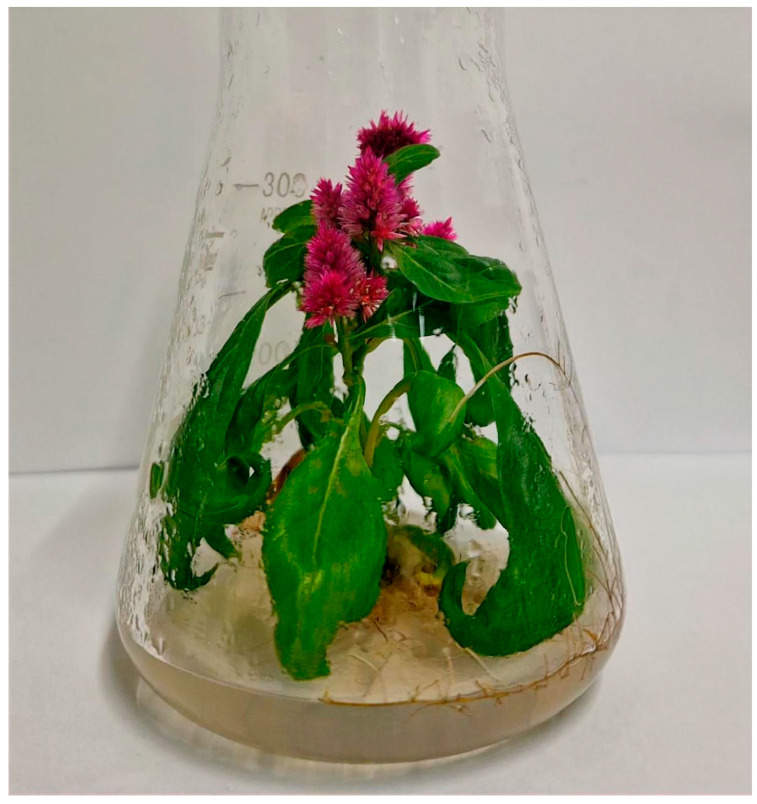
Flowering in vitro shoots of *Celosia* sp. (Photograph by M. Jeziorek; author’s own unpublished research).

Research on amaranthin in plant in vitro cultures has emerged as a critical area of inquiry due to the multifaceted potential significance of this betacyanin pigment in the nutrition, medicine, and cosmetic industries. A summary of experimental studies on the biosynthesis of amaranthin in organ and tissue in vitro cultures is presented below in Table 3. Presenting the predominant plant in vitro systems (shoot cultures and callus and suspension cultures), it also outlines various research foci within the still-developing multi-directional trend of identifying efficient methods of obtaining valuable plant metabolites through biotechnological approaches. Figure 3 shows *Celosia* sp. shoots obtained from seeds on solid culture medium (based on the author’s own unpublished research).

### 5.1. Diverse In Vitro Culture Platforms

Researchers have employed a wide array of in vitro systems—callus cultures, cell suspensions, organogenic shoots, immobilized beads and even heterologous tobacco bright yellow-2 (BY-2) line—to investigate and enhance betalain biosynthesis. Early foundational work by Berlin et al. (1986) focused on *Chenopodium rubrum* suspension cultures as a reference system [87], while later studies extended to calli of *Amaranthus tricolor* [84], *Alternanthera* spp. [81,82,83] and *Celosia* spp. [56,85,86]. Immobilization (alginate–chitosan beads) and physical permeabilization (electric fields, high pressure) of *C. rubrum* cells were introduced as green extraction methods to recover intracellular amaranthin with up to 85% release efficiencies by Dörnenburg and Knorr (1993) [89]; analogous chitosan/DMSO treatments used by Knorr and Berlin (1987) also liberated pigment but revealed rapid degradation if not stabilized [92].

It is worth noting the successful attempts at genetic transformation to intensify amamrantin production [93,94]. One of the recent advances reconstitutes the amaranthin pathway in tobacco BY-2 cells by over-expressing four *Chenopodium quinoa* genes, including the newly isolated amaranthin synthetase. This chassis produced 13.7 µM amaranthin and 26.6 µM betanin, demonstrating proof-of-concept for microbial-style pigment factories [93]. Glitz et al. (2025) successfully engineered yeast cell factories for recombinant amaranthin production, achieving remarkable yields of 2.97 g L^−1^ ± 29.3 mg L^−1^ in fed-batch fermentation using *Yarrowia lipolytica* strains equipped with glucuronosyltransferases and UDP-glucose dehydrogenase from *Arabidopsis thaliana* for de novo UDP-glucuronic acid synthesis [94]. Hairy root in vitro cultures of *Beta vulgaris* have additionally proved to be a system of betalains production, which justifies undertaking similar attempts to obtain amaranthin from species in which it naturally occurs [95,96].

### 5.2. Media Composition and Precursor Feeding

Most studies utilize Murashige and Skoog (MS) basal salts, originally described in 1962, with variations in sucrose (3–6%), auxins [2,4-dichlorophenoxyacetic acid (2,4-D) and 1-naphthaleneacetic acid (NAA)], cytokinins [kinetin, 6-benzylaminopurine (BAP), and thidiazuron (TDZ)] and, increasingly, precursor feeds such as L-tyrosine. Tyrosine feeding emerged as a simple yet effective booster in *C. rubrum* suspensions—raising total betacyanins to ~100 mg L^−1^ (~1% DW), with amaranthin comprising ~80% of the pool (Berlin et al., 1986) [87]—and in *Alternanthera* shoots, delivering up to 51 mg 100 g^−1^ FM (Kleinowski et al., 2014) [82]. Carbon-source trials in *Celosia* calli revealed that sucrose remains superior to hexoses for maximal pigment yield, while also guiding the discovery of malonylated amaranthin derivatives [56].

### 5.3. Elicitation and Light Quality

Beyond simple precursors, biotic elicitors and light spectra have been harnessed to tailor pigment output. Elicitation studies using methyl jasmonate in *Alternanthera sessilis* have demonstrated the potential to enhance bioactive compound accumulation through transcriptomic approaches [97]. A *Fusarium oxysporum* cell-wall lysate at 0.125 ‰ *w*/*v* significantly elevated both amaranthin and betanin in *Celosia cristata* suspensions, without growth penalties [86]. Regarding light quality, LED light treatments (red, white, and blue) applied to *Alternanthera* microshoots showed red/white light to be most stimulatory for betalain accumulation [83].

### 5.4. Enzymology and Biosynthetic Pathways

The molecular understanding of betalain biosynthesis has advanced through plant tissue culture studies, with research on *Amaranthus tricolor* elucidating core biosynthetic pathways and identifying key regulatory genes [6,98,99]. The transcriptomic and enzymatic analyses shedding light on the biosynthetic genes responsible for glycosylation and pigment accumulation in *Amaranthaceae* species direct the potential to increase bioactive compound production [6,97,98,99]. A parallel thread of Bokern et al. studies dissected the enzymatic machinery: crude protein preps from *Chenopodium rubrum* were shown to acylate amaranthin to celosianin I/II [100], and the responsible hydroxycinnamoyl transferase was purified ~500-fold, characterized kinetically [91]. Growth-curve studies further correlated transferase activity with temporal betacyanin buildup [90].

The comparative analysis reveals that while plant in vitro systems have provided crucial foundational knowledge for understanding amaranthin biosynthesis and have demonstrated various production approaches, engineered yeast cell factories currently represent a more efficient and scalable platform for amaranthin production. The significantly higher yields achieved through microbial fermentation, combined with the stability and industrial scalability of yeast systems, position recombinant production as the preferred method for commercial amaranthin production in biotechnological applications.

## 6. Regulation of Amaranthin Biosynthesis

### 6.1. Amaranthin Biosynthetic Pathway

**Figure 4:**
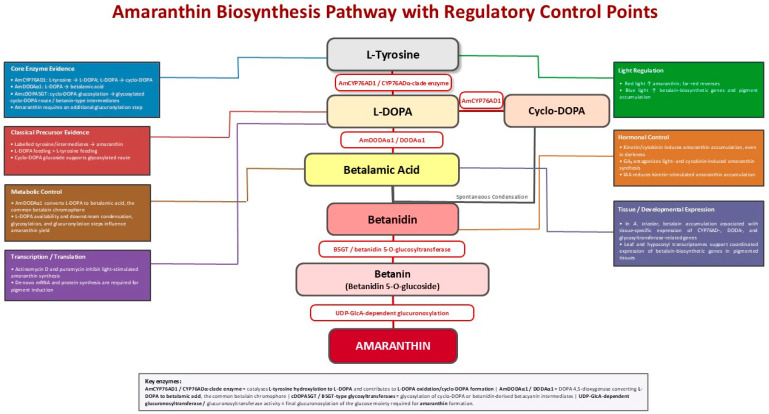
Proposed biosynthetic pathway and regulatory control of amaranthin.

The amaranthin biosynthetic pathway indicating the key enzymes involved in the process is presented in Figure 4.

In betalain-producing *Amaranthus* species, amaranthin biosynthesis proceeds through the general betacyanin pathway from the aromatic amino acid L-tyrosine (Figure 4). Tyrosine is first hydroxylated to 3,4-Dihydroxy-L-phenylalanine (L-DOPA) by CYP76AD-type cytochrome P450 enzymes, which may also participate in the formation of cyclo-3,4-dihydroxyphenylalanine (cyclo-DOPA). L-DOPA can be cleaved by DOPA 4,5-dioxygenase (DODA) to produce betalamic acid, the chromophoric precursor common to all betalains, while a parallel oxidation step yields cyclo-DOPA. Condensation between betalamic acid and cyclo-DOPA gives rise to the betacyanin aglycone betanidin. Subsequent 5-O-glucosylation of betanidin and glucuronylation at the glucose moiety result in the formation of amaranthin (betanidin 5-O-[β-D-glucuronosyl-(1→2)-β-D-glucoside]). This sequence has been substantiated by classical feeding experiments with labeled tyrosine and intermediates in *Amaranthus*, as well as by the molecular characterization of *CYP76AD*, *DODA*, and glucosyltransferase genes in beet and amaranth [6,18,101,102,103].

It was proven that in *Amaranthus* spp., amaranthin synthesis is tightly regulated by light, particularly through the phytochrome system, where red light stimulates pigment production and far-red light reverses this effect [104]. Blue and UV light further enhance amaranthin biosynthesis via cryptochrome activation, revealing light-quality-dependent regulation [105]. Pigment accumulation can also be induced in complete darkness with exogenous kinetin, a cytokinin that activates transcription of the biosynthetic enzymes [106,107]. However, gibberellic acid (GA_3_) antagonizes this effect, inhibiting both light- and cytokinin-induced synthesis, likely by modulating precursor allocation or repressing gene expression [108,109]. The need for active transcription and translation was demonstrated by the complete inhibition of pigment synthesis by actinomycin D and puromycin [110,111]. DOPA and tyrosine feeding studies confirmed the importance of precursor supply, with DOPA being more effective and light-enhanced, which indicates that a key enzymatic conversion step is light-dependent [112]. Further biochemical elucidation in *Celosia plumosa* showed that cyclo-DOPA and its glucoside serve as more efficient amaranthin precursors than betanidin or betanin, validating the specific biosynthetic route [113]. Cyclic nucleotides such as dibutyryl cyclic adenosine monophosphate (db-cAMP) and N^6^-substituted adenines also stimulated pigment production but lacked an amplifying effect with light, implying that they mimic cytokinins rather than act as second messengers in light pathways [114]. This type of experimental system is being continued. Similarly, Zhu et al. (2016) performed experiments based on methods developed by Köhler et al. (1980) and, with minor modifications, investigated the antagonistic effect of indole-3-acetic acid on kinetin-stimulated amaranthin accumulation in the cotyledons of *Amaranthus mangostanus* seedlings [115,116]. Transcriptomic analyses of *Amaranthus tricolor* tissues performed by Liu et al. (2019) and Liu et al. (2023) provided additional insights into betalain biosynthesis and regulation [99,117]. Together, these studies generated a detailed catalog of genes and a tissue-specific expression map for betalain biosynthesis in *A. tricolor* and showed that environmental cues such as blue light influence pigment accumulation by increasing the transcription of biosynthetic genes, likely through a GA–DELLA-associated regulatory pathway.

### 6.2. Classical Studies on Hormonal and Physical Factors Influencing Amaranthin Biosynthesis

This section reviews noteworthy studies conducted on seedlings of *Amaranthaceae* plants and examining amaranthin biosynthesis, including the influence of physical factors and growth regulators. The findings derived from these investigations, predominantly conducted during the 1970s and early 1980s, established a fundamental comprehension of the influences exerted by variables such as light and kinetin on the biosynthesis of amaranthin within plant tissues [110,111,112]. This segment of classical research was focused on species such as *Amaranthus caudatus*, *A. tricolor*, and also *Celosia plumosa* [108,109,110,113].

The consistent applied methodology enabled precise control of the experimental variables and ensured comparability across light and hormone treatment regimes. Seedlings were cultivated under specific controlled laboratory conditions: seeds were typically germinated on moistened filter paper in Petri dishes and maintained in darkness at temperatures ranging from 25 to 28 °C. After an initial dark growth period (48–72 h), seedlings were exposed to various light treatments (white, red, far-red, blue, UV) or incubated with hormonal, nucleotide, or biochemical precursor solutions.

Collectively, the studies affirm that amaranthin biosynthesis is governed by light-dependent photoreceptors, hormone signaling, and transcriptional activity, offering a detailed model for pigment regulation in plants. The experimental consistency and physiological relevance of these findings positioned *Amaranthus* sp. as providing a cornerstone system for exploring light–hormone interactions in secondary metabolism.

## 7. Biological Activity of Amaranthin

The literature on the biological activity of amaranthin highlights several major themes, mainly its antioxidant and anti-inflammatory properties. Emerging studies have examined molecular mechanisms, bioavailability, and potential therapeutic applications, illustrating a growing integration of biochemical, pharmacological, and food science perspectives.

### 7.1. Antioxidant Properties

Amaranthin and related betacyanins exhibit strong antioxidant activity, efficiently scavenging free radicals such as DPPH, hydroxyl radicals, and superoxide anions. Multiple studies consistently demonstrate the strong antioxidant and radical scavenging activities of amaranthin-containing extracts, with evidence of effective quenching of reactive oxygen species and free radicals [62,63,80,118,119,120]. Comparative analyses performed by Fernando et al. (2023) indicated that betacyanins exhibit superior radical scavenging compared to betaxanthins [120]. Associations between pigment content and DPPH- and ABTS-based radical scavenging capacity have been reported in various *Amaranthus* genotypes by Sarker et al. (2020) [11], Sarker et al. (2021) [2], and Sarker et al. (2022) [119,121].

Amaranthin and isoamaranthin exhibit antioxidant activity through radical scavenging and cellular redox modulation, though with lower potency than other betacyanins. In comparative DPPH assays, amaranthin/isoamaranthin ranked among the least active betalains tested (EC50 ≈ 8.0 μM), approximately 2.2-fold less potent than gomphrenins (EC50 ≈ 3.7 μM) and 1.5-fold less active than betanin (EC50 ≈ 5.5 μM) [62]. *Amaranthus caudatus* flower extracts rich in amaranthin (171 mg/g of extract) and isoamaranthin (38 mg/g of extract) demonstrated OxHLIA IC50 values of 29.0 μg/mL (60 min) [63]. At the cellular level, amaranthin-type betacyanins protected H9c2 cardiomyoblasts against H2O2-induced damage at 0.1–10 μg/mL, increasing intracellular glutathione levels [45,61].

### 7.2. Anti-Inflammatory Effects

Betalains demonstrate anti-inflammatory activity by suppressing pro-inflammatory cytokines such as interleukin-6 (IL-6) and tumor necrosis factor-alpha (TNF-α), and enzymes, including inducible nitric oxide synthase (iNOS) and cyclooxygenase-2 (COX-2), thereby modulating immune responses in vitro and in vivo. Evidence from cellular and animal models supports their anti-inflammatory potential, including suppression of pro-inflammatory cytokines and enzymes, modulation of signaling pathways such as MAPK/NF-κB, and enhancement of antioxidant defenses [120,122]. Betalain extracts have been shown to reduce leukocyte recruitment and oxidative stress markers in inflammation models, which suggests their therapeutic relevance [123].

*Atriplex hortensis* extracts and purified amaranthin-type pigments reduced prostaglandin E2 (PGE2) production in LPS-stimulated RAW264.7 macrophages [75]. Tyszka-Czochara et al. (2016) also investigated selenium supplementation and reported that amaranth sprouts with the highest amount of betacyanins (19.30 ± 0.57–28.85 ± 2.23 mg of amaranthin/100 g of fresh weight) and a high total selenium content exerted an anti-inflammatory effect, decreasing inflammatory interleukin-6 production in activated RAW264.7 macrophages [124].

### 7.3. Antimicrobial and Antiviral Activities Studies

Amaranthin has been investigated for its antimicrobial activity primarily as a component of betacyanin-rich fractions rather than as an isolated compound. Studies by Yong et al. demonstrated that betacyanin fractions from red spinach (*Amaranthus dubius*) and red pitahaya (*Hylocereus polyrhizus*) exhibited inhibitory effects against *Staphylococcus aureus* and *Pseudomonas aeruginosa*, including biofilm formation on various polymer surfaces [72,125,126]. Comparative analyses indicated stronger antimicrobial activity of red spinach betacyanin fractions, which contain amaranthin-type pigments, in planctonic assays [72]. However, anti-biofilm activity was pathogen-dependent, with red spinach fractions showing stronger inhibition against *P. aeruginosa* biofilms [125]. These findings suggest that amaranthin-type betacyanins may contribute to the observed antimicrobial effects in red spinach fractions, though the specific contribution of amaranthin versus other betacyanins in the fractions remains to be fully elucidated.

Recent investigations have expanded the bioactivities of betacyanin fractions to include antiviral activity. Lim et al. (2024) reported inhibitory effects of red pitahaya betacyanin fractions against the influenza A virus [127], while Chang et al. demonstrated the antiviral activity of betacyanin fractions from both red pitahaya and red spinach against dengue virus type 2 [71].

The variability in reported antimicrobial efficacy may be attributed to several factors, including differences in betacyanin fraction composition, microbial strain susceptibility, and assay methodologies. The enhanced biofilm-inhibiying activity observed for combined red spinach and red pitahaya betacyanin fractions compared with single-source fractions suggests possible additive or synergistic effects among fraction constituents; however, the specific contribution of amaranthin was not determined [126].

### 7.4. Comparative Bioactivity with Other Betacyanins

Comparative analyses reveal that amaranthin shares many bioactivities with other betacyanins, but they may differ in potency and mechanism. Amaranthin and isoamaranthin demonstrate measurable antioxidant, anti-inflammatory, and cardioprotective activities at low micromolar concentrations, though they are less potent than betanin or gomphrenins in direct assays [45,61,62,63,75]. Amaranthin’s biological effects involve ROS scavenging, glutathione elevation, NF-κB inhibition, and pro-inflammatory mediator suppression [45,61,75,124]. Most anti-inflammatory studies focus on betanin or mixed betalain extracts rather than isolated amaranthin, which limits direct attribution of effects. More specific data for isolated amaranthin is still in demand, and the dose–response relationships are also to be well established [120,122,123,128,129]. The lower antioxidant potency is attributed to glycosylation patterns and hydroxyl group positioning that reduce hydrogen-donation capacity compared to more active betalains [62]. Direct comparative studies specifically including amaranthin are limited, and many focus on betanin or other betacyanins from different plant sources, which complicates extrapolation [62,130]. The diversity of betacyanin structures and their derivatives necessitates more systematic studies of structure–activity relationships to clarify differences [61,120].

Research gaps include: (i) the absence of in vivo data for purified compounds, (ii) limited bioavailability studies, and (iii) insufficient evidence for neuroprotective, hepatoprotective, or anticancer activities. Despite a lower per-molecule potency, amaranthin’s abundance in *Amaranthus* species and synergistic contributions to whole-food bioactivity warrant further investigation, particularly regarding oxidized derivatives that may exhibit enhanced biological activities [45,61]. Mentioned previously innovative extraction techniques such as microwave-assisted and ultrasound-assisted methods have improved amaranthin recovery and purity and, thus, may also enable reliable bioactivity research [63,79,80].

Some studies have begun to elucidate the molecular pathways affected by amaranthin and related betacyanins, including modulation of oxidative stress enzymes, inflammatory mediators, and apoptotic proteins [120,122,128,129]. Detailed mechanistic studies remain scarce, particularly for amaranthin specifically. The complexity of cellular responses and the interplay with other phytochemicals complicate interpretation. Data derived from in vitro or animal models still have limited translation to human physiology [120,129]. The bioavailability and metabolism of amaranthin in vivo are not well characterized, which hinders our understanding of its systemic effects, and the impact of oxidation and degradation products on bioactivity is not fully understood [61,129].

There is a scarcity of animal models or human clinical studies evaluating the efficacy and safety of amaranthin, which limits the evidence base for its therapeutic potential and external validity in real-world applications [61,93,122]. Most research focuses on antioxidant and anti-inflammatory properties, with less attention to other bioactivities such as antiviral, cytoprotective, or metabolic effects, which restricts the comprehensive understanding of amaranthin’s full biological profile. Although some areas require further investigation, current research identifies amaranthin as a potentially valuable bioactive compound worth detailed focus not only as a natural food and cosmetic colorant but also in terms of its nutraceutical and possible therapeutic applications.

## 8. Practical Implications

Together with the aspects mentioned earlier, there are multiple areas of developing application potential for amaranthin and amaranthin-like natural pigments. To name some of the significant ones, a few threads from this area are pointed out below.

The strong antioxidant and anti-inflammatory properties of amaranthin suggest its potential application as a natural therapeutic agent or dietary supplement for managing oxidative stress-related and inflammatory diseases, supporting its development in the nutraceutical and pharmaceutical industries [120,122,123]. Amaranthin-rich extracts have already been initially tested as natural food colorants in various matrices including yogurt, pasta, and confectionery [13,47,131]. The functional benefits, including antioxidant and antimicrobial activities, can additionally enhance the final product value [4,47]. The demonstrated antimicrobial and anti-biofilm activities of amaranthin-containing extracts against clinically relevant pathogens highlight their potential for incorporation into food preservation systems and medical device coatings to reduce infection risks [71,125,126].

Advances in extraction and analytical techniques, including ultrasound-assisted and microwave-assisted methods, improve the yield, purity, and stability assessment of amaranthin, facilitating its practical use as a natural colorant and bioactive ingredient in food and cosmetic products [63,79,80].

Insights into the biosynthetic genes and enzymatic pathways responsible for amaranthin production enable biotechnological approaches such as transgenic cell cultures for scalable pigment production, which can meet industrial demands for natural colorants and bioactives [92,97].

The identification of amaranthin’s antiviral effects against influenza A virus and its non-cytotoxic profile in vitro suggest promising avenues for developing plant-based antiviral agents, which is particularly relevant for public health and pharmaceutical innovation [127].

The correlation between pigment content, antioxidant capacity, and environmental factors such as light exposure informs agricultural practices and cultivar selection for optimizing amaranthin yield and bioactivity, benefiting functional food development and crop improvement programs [2,11,116,119,121].

Amaranthin is also known for its application in the “amaranthin reduction test” in plant-focused research. This method relies on the pigment production in seedlings of *Amaranthus* under normal conditions. When gibberellins (such as GA_3_) are applied, they inhibit the synthesis of amaranthin. By measuring the decrease in its content (usually via spectrophotometry), researchers can estimate the gibberellin activity or concentration in a sample [132]. Additionally, not directly amaranthin-dependent but based on betalain synthesis and worth mentioning is the RUBY reporter system developed for gene expression and transformation monitoring in plants. RUBY is a genetic construct/cassette containing three betalain-biosynthetic genes, *CYP76AD1*, *DODA*, and glucosyltransferase, originally derived from *Beta vulgaris*. When introduced into a target plant’s genome during transformation, it triggers production of a vivid red-purple pigment in the tissues where it is expressed. Because betalains are not normally present in most plant species, pigment accumulation is a clear, easily visible indicator that transformation was successful—no staining, microscopy, or chemical assays required [133].

## 9. Conclusions

The integrated evidence from the studies reviewed provides an updated perspective on amaranthin as a betacyanin pigment with multifunctional potential. This review focuses on its plant sources and also summarizes in vitro production systems, as well as the analytical methods used for its identification and quantification, extraction protocols, and currently reported biological activities. Among natural sources, cultivated *Amaranthus* species demonstrate significantly higher amaranthin concentrations than wild species, with total betacyanin contents reported to range from 46.1 to 199 mg/100 g fresh weight, and amaranthin comprising, on average, 80.9% of the betacyanin fraction [7]. With regard to in vitro culture techniques, the review indicates that selected in vitro plant tissues and transgenic organisms with elevated amaranthin concentrations could serve as viable alternatives to synthetic colorants, although specific quantitative data for the highest-yielding systems would require further detailed analysis of the sections discussing tissue culture methodologies. Collectively, the reviewed studies identify *Amaranthus* sp. (cultivated), *Chenopodium* sp. (in vitro suspensions), and *Alternanthera* sp. (in vitro shoots) as the three most productive genera for amaranthin biosynthesis, with additional contributions from *Celosia* sp., *Atriplex* sp., and genetically engineered *Nicotiana* sp. systems. The highest reported amaranthin titer to date was achieved through microbial fermentation, reaching 2.97 ± 0.029 g L^−1^ in fed-batch cultivation of engineered *Yarrowia lipolytica* strain ST14102 expressing CcAmaSy1 [94]. Together with the robustness and scalability of yeast platforms, these results support recombinant production as a promising route toward biotechnology-oriented amaranthin manufacturing.

These studies indicate that amaranthin is not only a promising natural pigment, but also a valuable model compound for integrating betalain biosynthesis, secondary metabolite bioactivity, and scalable production strategies for industrially relevant natural products. Selected areas of significance are highlighted below.

### 9.1. Development of Betacyanin Extraction

The extraction of betacyanins (including amaranthin and isoamaranthin) from plant materials has evolved significantly from traditional aqueous and alcoholic solvent methods toward advanced green extraction techniques, with ultrasound-assisted extraction optimized through response surface methodology representing the current state-of-the-art, achieving amaranthin concentrations of 171 ± 1 mg/g extract (isoamaranthin at 38 ± 1 mg/g extract) from *Amaranthus caudatus* flowers [63]. Traditional methods employing water, methanol, or aqueous ethanol remain widely used, with reported amaranthin yields ranging from 19.30 to 28.85 mg/100 g fresh weight (FW) in sprouts [124], though these conventional approaches typically require longer extraction times (1–24 h) and may result in lower efficiency compared to assisted extraction methods [134,135]. Biotechnological production through engineered cell suspension cultures represents an emerging alternative, with transgenic tobacco BY-2 cells producing amaranthin at 13.67 ± 4.13 μM, which demonstrates the feasibility of in vitro biosynthesis independent of agricultural constraints [93].The field has witnessed a clear trend toward sustainability and process intensification, with recent studies emphasizing green chemistry principles, reduced solvent consumption, and systematic optimization using design of experiments (DoE) approaches [63,134], while advanced analytical techniques including LC-Orbitrap-MS and NMR have enabled precise structural characterization of amaranthin-type betacyanins and their oxidized derivatives [45,61]. Key optimization parameters include pH (optimal 3–5), temperature (35–45 °C), ultrasound power, and solid-to-liquid ratio, with acidified solvents enhancing both extraction efficiency and pigment stability [63,134,135].

### 9.2. Current Research Domains

The available data position amaranthin as a versatile plant-derived betacyanin with multiple functional roles and a broad range of potential applications. Beyond its role as a natural pigment, amaranthin is increasingly considered both as a natural colorant and as a bioactive compound, which supports its relevance for food, cosmetic, nutraceutical, and biotechnological applications. Current research is particularly concentrated in several areas:

- Commercial colorant development: studies assessing *Amaranthus* species and related sources as natural colorants [1,7,12,136];
- Bioactivity assessment: investigations of antioxidant and other health-related properties of amaranthin-rich extracts and amaranthin-type pigments [2,65];
- Extraction optimization: studies aimed at improving extraction efficiency, pigment recovery, and stability [47,63];
- Genetic and biosynthetic pathways: research on betalain biosynthesis, pathway enzymes, and regulatory mechanisms involved in amaranthin production [24].

### 9.3. Priority Areas for Future Investigation

This analysis highlights several priority areas for future research, including: (i) harmonization of extraction, quantification, and reporting protocols for amaranthin to enable cross-study comparisons; (ii) systematic screening and further genetic or biotechnological improvement of both field-grown and in vitro sources to obtain high and stable pigment production; (iii) detailed structure–stability–bioavailability–activity studies, particularly in relevant in vivo models, to underpin health-related claims; and (iv) translational research on formulation, processing stability, and safety to support regulatory approval and industrial implementation. Above all, the stability of amaranthin under various processing conditions remains a critical factor influencing its bioavailability and broader application potential. Therefore, this area is likely to progress rapidly with more detailed and targeted studies.

## Figures and Tables

**Table 1:** Various uses and related contexts of ‘amaranthin’ term in scientific literature.

‘Amaranthin’ Type Characteristics	Short Description of Research Area	Example Study Focus—Field of Research	Ref.
Natural pigment of plant origin (betacyanin)	Plant physiology, food coloration, antioxidants	Optimization of extraction from *Amaranthus hypochondriacus*	[28]
Lectin protein	Plant defense, lectin binding studies	T-antigen recognition; binding studies in human colon tissue	[29,30]
Storage protein (11S globulin)	Nutraceutical enhancement, antihypertensive peptide expression	Transgenic maize and tobacco cells expressing amarantin	[31,32]
Amaranth red (E 123—synthetic pigment)	Food and cosmetic pigment (modified red azo dye)	Detection using sensor materials	[40,41,42]

**Table 3:** Comparative Overview of Amaranthin-Focused In Vitro Systems.

Plant or Yeast Species	In Vitro Culture Characteristics	Aim of the Study	Culturing Conditions	Principal Findings	Investigated Tissue	Amaranthin Quantity	Ref.
*Alternanthera* *brasiliana* (L.) Kuntze	Magenta callus	Develop reliable callus system and quantify pigments by HPLC-MS/MS	MS + phytagel 0.2%; 30 g L^−1^ sucrose + 100 mg L^−1^ inositol + AA 3 mg L^−1^; best callus induction medium (CIM) = 0.75 mg L^−1^ IAA + 1 mg L^−1^ 2,4-D; 22 d dark + 8 d light	Internode calli on CIM3 gave 262 µmol g^−1^ DM * amaranthin; leaf calli lower	Callus	262 µmol g^−1^ DM	[81]
*Alternanthera* *philoxeroides* (Mart.) Griseb., *A. tenella* Colla	In vitro shoots	Assess precursor feeding on pigment and growth	MS + 0–75 µM L-tyrosine; 45 d, 16 h light	Best betacyanin 51.3 mg 100 g^−1^ FM * in *A. philoxeroides* stems at ~45 µM tyrosine; 36.5 mg 100 g^−1^ FM in *A. tenella* shoots	Shoots	36–51 mg 100 g^−1^ FM	[82]
*Alternanthera* spp. (*A. sessilis*, *A. philoxeroides*, *A. tenella*, *A. brasiliana*)	Whole plant microshoots	Light quality effects on pigment profiles	MS; 45 d under monochromatic LEDs (blue, red, white)	Red and white LEDs boosted betacyanins in all species; red light maximized biomass	Shoots	n.g. ** (relative growth noted)	[83]
*Amaranthus tricolor* L.	Light-responsive suspension line	Establish single pigment (amaranthin) model; study light vs. cytokinin synergy	MS + 0.15 µM 2,4-D + 1 µM kinetin; 16 h light; 3% sucrose	Peak production ~30 µmol g^−1^ DW * (day 68); amaranthin: iso 10:1	Suspension biomass	~30 µmol g^−1^ DW	[84]
*Amaranthus tricolor* L.	Stable red callus line	Generate furnishing grade colorant callus	MS + 0.25 mg L^−1^ NAA + 2 mg L^−1^ BAP; dark	Deep-red callus with 12 mg g^−1^ DW betacyanins (amaranthin dominant)	Callus	12 mg g^−1^ DW	[9]
*Celosia argentea* L. var. *plumosa*	Optimized suspension culture	Response surface methodology (RSM) optimization for commercial pigment yields	MS + 43.9 g L^−1^ sucrose + 0.77 mg L^−1^ BAP + 0.15 mg L^−1^ tyrosine; 15 d, 110 rpm	Peak 43.9 mg L^−1^ total betalains	Suspension biomass	n.g.	[85]
*Celosia cristata* L.	Cell suspension (magenta callus-derived)	Biotic/abiotic elicitors impact for betalain boost	MS + 13.5 µM 2,4-D + 0.44 µM BAP; elicited at log phase with 0.125 ‰ *Fusarium oxysporum* lysate	0.125 ‰ fungal elicitor significantly increased (almost 2-fold) content of amaranthin, betanin, betalamic acid and betaxanthin	Suspension biomass	n.g.	[86]
	Intensely red callus	Profile and identify new malonyl amaranthins	MS + 30 g L^−1^ sucrose; variable carbon sources (sucrose, maltose, fructose, glucose)	Amaranthin major betacyanin; novel 6′Omalonylamaranthin isolated; betalain:betaxanthin ≈ 10:1	Red callus	Up to 66.2% of total betacyanins	[56]
*Chenopodium* *rubrum* L.	Photoheterotrophic cell suspension	Define a high-yield reference line for betalain production and medium optimization	Basal MS salts + 3% sucrose; continuous shake (120 rpm); 16 h light; optional 15 µM L-tyrosine feed	Tyrosine feeding + inoculum optimization raised betacyanins to ~1% DW (≈100 mg L^−1^); amaranthin 80% of pool	Suspension biomass	Up to 80% of all betacyanins	[87]
	Alginate–chitosan immobilized cells	Test immobilization/permeabilization for pigment release and cell viability	Same growth medium; beads cured in CaCl_2_ (1.5%) or KCl (1.5%); permeabilized with water-soluble chitosan (≤1 mg mL^−1^) or 10% DMSO	Water-soluble chitosan < 500 µg mL^−1^ released amaranthin with minimal viability loss; up to 18% of cellular pigment recovered	Immobilized cell beads	n.g	[88]
	Electropulsed/high-pressure suspension	Evaluate physical cell permeabilization for downstream recovery	MS + 0.4 mg L^−1^ 2,4-D; electric field 0 to 1.6 kV cm^−1^ (0 to 30 pulses) or hydrostatic pressure 0.1 to 350 MPa (10 min)	85% of total amaranthin released at 1.6 kV cm^−1^ but viability lost; 99% pigment release after 350 MPa	Cells; cells and medium	Up to 817 µg g^−1^ FW (cells); up to 195.7 mg L^−1^ (cells and medium)	[89]
	Deep-red CH/CHN suspension lines	Relate feruloylglucose metabolism to betacyanin accrual	MS medium + 3% sucrose; standard photomixotrophic culture	CHN line accumulated markedly higher amaranthin and celosianin II than CH; higher amaranthintransferase activity	Suspension cells	Relative growth (n.g.)	[90]
	Suspension (enzyme purification study)	Purify and characterize amaranthin O-acyltransferase activity	MS medium; cell-free extracts purified 515-fold	Vmax 910 pkat mg^−1^; specific for amaranthin vs. isoamaranthin	Purified enzyme	n.g.	[91]
	Chitosan/DMSO permeabilized suspension culture	Kinetics of amaranthin release/degradation post-treatment	MX-medium (MS + 2 µM 2,4-D) with added chitosan/DMSO/calcium alginate gel beads	Water-soluble chitosan gave 80% radiolabel release but only 19% intact amaranthin (rapid degradation)	Suspension cells + medium	Max. conc. 6.80 µg mL^−1^	[92]
*Nicotiana tabacum* L. BY-2 (transgenic)	Suspension cells expressing 4 quinoa genes	Reconstitute amaranthin biosynthesis in heterologous chassis	LS medium + 0.2 mg L^−1^ 2,4-D, 3% sucrose;	13.7 µM amaranthin + 26.6 µM betanin accumulated; amaranthin inhibited HIV-1 protease	BY-2 cells	13.67 ± 4.13 µM	[93]
*Saccharomyces* *cerevisiae* (engineered strains ST14115-ST14118)	Genetically modified yeast strains with integrated glucuronosyltransferases (GlcATs) and/or UDP-glucose dehydrogenase (AtUGD1)	Screen GlcATs for amaranthin formation and investigate UDP-glucuronic acid biosynthesis pathway	MM medium ± pABA; 24-well plates; 30 °C, 250 rpm, 48 h	Integration of AtUGD1 increased UDP-glucuronic acid pool to 34 µM; GlcAT + AtUGD1 strains produced amaranthin with visible pink-red coloration	Recombinant yeast cells	10.2 ± 2.7 mg L^−1^ (CqAmaSy1 strain/ST14116)	[94]
*Yarrowia* *lipolytica* (engineered strains ST14100-ST14102)	High-producing betanin strain engineered with GlcATs; small-scale screening; ST14102 used for fed-batch fermentation	Evaluate GlcAT performance and scale up amaranthin production in industrial yeast strain	MM medium; 24-well plates and AMBR bioreactors; exponential feeding at 0.1 h^−1^ specific growth rate	Amaranthin titers exceeded betanin by 1.6–3.9 fold; endogenous UDP-glucuronic acid synthesis capability; approx. threefold higher than the betanin reference fermentation	Recombinant yeast cells	2.97 ± 0.029 g L^−1^ (fed-batch fermentation of strain ST14102 expressing CcAmaSy1)	[94]

Notes. * DM/FM/DW—dry mass/fresh mass/dry weight, respectively, as reported in the cited studies. ** n.g., not given; quantitative value not reported. Where studies targeted total betalains, the dominant betacyanin was amaranthin unless otherwise stated.

## Data Availability

No new data were created or analyzed in this study.

## Abbreviations

The following abbreviations are used in this manuscript:

2,4-D	2,4-Dichlorophenoxyacetic acid
AA	Ascorbic acid
ABTS	2,2′-azino-bis(3-ethylbenzothiazoline-6-sulfonic acid)
AI	Artificial intelligence
AMBR	Automated micro-bioreactor system
ATR-FTIR	Attenuated total reflectance–Fourier transform infrared
AtUGD1	*Arabidopsis thaliana* UDP-glucose dehydrogenase 1
BAP	6-Benzylaminopurine
BY-2	Bright Yellow 2 (*Nicotiana tabacum* cell line)
db-cAMP	Dibutyryl cyclic adenosine monophosphate
CH and CHN	*Chenopodium rubrum* cell lines
C.I.	Color index
CIM	Callus induction medium
COX-2	Cyclooxygenase-2
cyclo-DOPA	Cyclo-3,4-dihydroxyphenylalanine
CYP76AD	Cytochrome P450 family 76 subfamily AD
DAD	Diode array detector
DE	Dry extract
DI-IM-MS	Direct infusion–ion mobility–mass spectrometry
DM	Dry mass
DMSO	Dimethyl sulfoxide
DODA	DOPA 4,5-dioxygenase
DoE	Design of experiments
L-DOPA	3,4-Dihydroxy-L-phenylalanine
DPPH	2,2-Diphenyl-1-picrylhydrazyl
DT	Dry tissue
DW	Dry weight
E123/E162	EU “E-number” of food colorants: E123, Amaranth synthetic azo dye; E162, Beetroot Red/betanin-rich beetroot extract
EC50	Half-maximal effective concentration
EDTA	Ethylenediaminetetraacetic acid
ESI	Electrospray ionization
EU	European Union
FDA	U.S. Food and Drug Administration
FM	Fresh mass
FTIR	Fourier-transform infrared spectroscopy
FW	Fresh weight
GA3	Gibberellic acid
GA–DELLA	Gibberellin–DELLA signaling pathway
GlcAT/GlcATs	Glucuronosyltransferase(s)
H9c2	Rat cardiomyoblast cell line
HIV-1	Human Immunodeficiency Virus type 1
HPLC	High-performance liquid chromatography
HSCCC	High-speed counter-current chromatography
IAA	Indole-3-acetic acid
IC50	Half-maximal inhibitory concentration
IL-6	Interleukin-6 (cytokine)
iNOS	Inducible nitric oxide synthase (enzyme)
IP-HSCCC	Ion-pair high-speed counter-current chromatography
LC	Liquid chromatography
LC-MS/MS	Liquid chromatography–tandem mass spectrometry
LED	Light-emitting diode
LPS	Lipopolysaccharide
LS	Linsmaier and Skoog plant tissue culture medium (1965)
LTQ-Orbitrap Elite MS	Linear trap quadrupole–Orbitrap Elite mass spectrometer
MALDI-QIT-TOF MS	Matrix-assisted laser desorption/ionization quadrupole ion trap time-of-flight mass spectrometry
MAPK	Mitogen-activated protein kinase
MAPK/NF-κB	Mitogen-activated protein kinase/Nuclear Factor kappa-light-chain-enhancer of activated B cells (signaling pathways)
MeOH/EtOH/H2O	Methanol/Ethanol/Water
MM	minimal/mineral medium
MS	Mass spectrometry/Murashige and Skoog plant tissue culture medium (1962)—context-dependent
NAA	1-Naphthaleneacetic acid
NF-κB	Nuclear Factor kappa-B
n.g.	Not given
NMR	Nuclear magnetic resonance
OxHLIA	Oxidative hemolysis inhibition assay
pABA	p-aminobenzoic acid
PAD or PDA	Photodiode array detector
PGE2	Prostaglandin E2
PLE	Pressurized liquid extraction
QTOF	Quadrupole time-of-flight (mass spectrometer)
RAW264.7	Murine macrophage cell line
ROS	Reactive oxygen species
RSM	Response surface methodology
RUBY	Betalain-based visible reporter system
SCC	Suspension cell culture
SPE	Solid-phase extraction
TDZ	Thidiazuron
TNF-α	Tumor necrosis factor alpha
TOF/Q-TOF MS	Time-of-flight/quadrupole time-of-flight mass spectrometry
UDP	Uridine diphosphate
UHPLC	Ultra-high-performance liquid chromatography
UV–Vis	Ultraviolet–visible
WAX	Weak anion exchange

## References

[1] Howard J.E., Villamil M.B., Riggins C.W. (2022). Amaranth as a natural food colorant source: Survey of germplasm and optimization of extraction methods for betalain pigments. Front. Plant Sci..

[2] Sarker U., Oba S. (2021). Color attributes, betacyanin, and carotenoid profiles, bioactive components, and radical quenching capacity in selected *Amaranthus gangeticus* leafy vegetables. Sci. Rep..

[3] Xu F., Yun D., Huang X., Sun B., Tang C., Liu J. (2023). Preparation, Characterization, and Application of pH-Response Color-Changeable Films Based on Pullulan, Cooked Amaranth (*Amaranthus tricolor* L.) Juice, and Bergamot Essential Oil. Foods.

[4] Sigwela V., De Wit M., du Toit A., Osthoff G., Hugo A. (2021). Bioactive betalain extracts from cactus pear fruit pulp, beetroot tubers, and amaranth leaves. Molecules.

[5] Packard E.E., Alves da Costa Ribeiro Quintans I.L., Adhikary D. (2021). Genetics of Betalain Pigments in Amaranth Species. The Amaranth Genome; Compendium of Plant Genomes.

[6] Chang Y.C., Chiu Y.C., Tsao N.W., Chou Y.L., Tan C.M., Chiang Y.H., Liao P.C., Lee Y.C., Hsieh L.C., Wang S.Y. (2021). Elucidation of the core betalain biosynthesis pathway in *Amaranthus tricolor*. Sci. Rep..

[7] Cai Y., Sun M., Wu H., Huang R., Corke H. (1998). Characterization and Quantification of Betacyanin Pigments from Diverse *Amaranthus* Species. J. Agric. Food Chem..

[8] Biswas M., Dey S., Sen R. (2013). Betalains from *Amaranthus tricolor* L.. J. Pharmacogn. Phytochem..

[9] Biswas M., Das S.S., Dey S. (2013). Establishment of a stable *Amaranthus tricolor* callus line for production of food colorant. Food Sci. Biotechnol..

[10] Shcherban A.B. (2021). Physiological, biochemical and genetic bases of amaranth (*Amaranthus* L.) breeding for food and feed purposes (a review). Proc. Appl. Bot. Genet. Breed..

[11] Sarker U., Oba S. (2020). Leaf pigmentation, its profiles and radical scavenging activity in selected *Amaranthus tricolor* leafy vegetables. Sci. Rep..

[12] Cai Y.-Z., Sun M., Corke H. (2005). Characterization and application of betalain pigments from plants of the Amaranthaceae. Trends Food Sci. Technol..

[13] Roriz C.L., Carocho M., Alves M.J., Rodrigues P., Morales P., Ferreira I.C.F.R., Heleno S.A., Barros L. (2023). Betacyanins obtained from alternative novel sources as natural food colorant additives: Incorporated in savory and sweet food products. Food Funct..

[14] Pavokovi D., Krsnik-Rasol M. (2011). Complex biochemistry and biotechnological production of betalains. Food Technol. Biotechnol..

[15] Chong P.H., Yusof Y.A., Aziz M.G., Mohd Nazli N., Chin N.L., Syed Muhammad S.K. (2014). Evaluation of solvent extraction of *Amaranth betacyanins* using multivariate analysis. Int. Food Res. J..

[16] Khan M.I., Giridhar P. (2015). Plant betalains: Chemistry and biochemistry. Phytochemistry.

[17] Gandía-Herrero F., García-Carmona F. (2013). Biosynthesis of betalains: Yellow and violet plant pigments. Trends Plant Sci..

[18] Polturak G., Aharoni A. (2018). “La Vie en Rose”: Biosynthesis, Sources, and Applications of Betalain Pigments. Mol. Plant.

[19] Araujo-León J.A., Aguilar-Hernández V., del Pino I.S., Brito-Argáez L., Peraza-Sánchez S.R., Xingú-López A., Ortiz-Andrade R. (2023). Analysis of Red Amaranth (*Amaranthus cruentus* L.) Betalains by LC-MS. J. Mex. Chem. Soc..

[20] López M.A.H., Luna-Suárez S., Macuil R.J.D., Cárdenas F.d.F.R. (2023). Simple and efficient protocol for amaranth betalains extraction and stability analysis by ATR-FTIR spectroscopy. J. Cereal Sci..

[21] Cai Y., Sun M., Corke H. (2005). HPLC characterization of betalains from plants in the Amaranthaceae. J. Chromatogr. Sci..

[22] Gins M.S., Gins V.K., Motyleva S.M., Kulikov I.M., Medvedev S.M., Pivovarov V.F., Mertvishcheva M.E. (2017). Metabolites with antioxidant and protective functions from leaves of vegetable amaranth (*Amaranthus tricolor* L.). Agric. Biol..

[23] Wang H., Xu D., Wang S., Wang A., Lei L., Jiang F., Yang B., Yuan L., Chen R., Zhang Y. (2022). Chromosome-scale *Amaranthus tricolor* genome provides insights into the evolution of the genus *Amaranthus* and the mechanism of betalain biosynthesis. DNA Res..

[24] Winkler T.S., Vollmer S.K., Dyballa-Rukes N., Metzger S., Stetter M.G. (2024). Isoform-resolved genome annotation enables mapping of tissue-specific betalain regulation in amaranth. New Phytol..

[25] Yang R., Huang T., Song W., An Z. (2023). Identification of WRKY gene family members in amaranth based on a transcriptome database and functional analysis of AtrWRKY42-2 in betalain metabolism. Front. Plant Sci..

[26] Vara D., De J.P., Gonza L., Casique-arroyo G., Martı N. (2014). Betacyanin Biosynthetic Genes and Enzymes Are Differentially Induced by (a)biotic Stress in *Amaranthus hypochondriacus*. PLoS ONE.

[27] Amaranthin 2D-Structure (PubChem). https://pubchem.ncbi.nlm.nih.gov/compound/Amaranthin#section=2D-Structure.

[28] Tabio-García D., Paraguay-Delgado F., Sánchez-Madrigal M., Quintero-Ramos A., Espinoza-Hicks J.C., Meléndez-Pizarro C.O., Ruiz-Gutiérrez M.G., Espitia-Rangel E. (2021). Optimisation of the ultrasound-assisted extraction of betalains and polyphenols from *Amaranthus hypochondriacus* var. Nutrisol. Ultrason. Sonochem..

[29] Rinderle S.J., Goldstein I.J., Matta K.L., Ratcliffe R.M. (1989). Isolation and characterization of amaranthin, a lectin present in the seeds of *Amaranthus caudatus*, that recognizes the T- (or cryptic T)-antigen. J. Biol. Chem..

[30] Sata T., Roth J., Zuber C., Stamm B., Rinderle S.J., Goldstein I.J., Heitz P.U. (1992). Studies on the Thomsen-Friedenreich antigen in human colon with the lectin Amaranthin: Normal and neoplastic epithelium express only cryptic T antigen. Lab. Investig..

[31] Reyes-Moreno C., Ayala-Rodríguez A.E., Milán-Carrillo J., Mora-Rochín S., López-Valenzuela J.A., Valdez-Ortiz A., Paredes-López O., Gutiérrez-Dorado R. (2013). Production of nixtamalized flour and tortillas from amarantin transgenic maize lime-cooked in a thermoplastic extruder. J. Cereal Sci..

[32] Santos-Ballardo D.U., Germán-Báez L.J., Cruz-Mendívil A., Fuentes-Gutiérrez C.I., Milán-Carrillo J., Reyes-Moreno C., Valdez-Ortiz A. (2013). Expression of the acidic-subunit of amarantin, carrying the antihypertensive biopeptides VY, in cell suspension cultures of *Nicotiana tabacum* NT1. Plant Cell Tissue Organ Cult..

[33] Santos-Ballardo D.U., Germán-Báez L.J., Ambriz-Pérez D.L., Meza-Ayala K.A., Luna-Avelar K.D., Valdez-Ortiz A. (2019). Optimizing the particle bombardment conditions in cell suspension cultures of *Nicotiana tabacum* and expression of the recombinant antihypertensive amarantin. S. Afr. J. Bot..

[34] (2024). Amaranth(E123) Market Report. https://www.verifiedmarketreports.com/product/amaranth-e123-market/.

[35] Downham A., Collins P. (2000). Colouring our foods in the last and next millennium. Int. J. Food Sci. Technol..

[36] European Food Safety Authority (EFSA) (2010). Scientific Opinion on the re-evaluation of Amaranth (E 123) as a food additive. EFSA J..

[37] Small G.R., Ruddy T.D. (2011). PET imaging of aortic atherosclerosis: Is combined imaging of plaque anatomy and function an amaranthine quest or conceivable reality?. J. Nucl. Cardiol..

[38] Wu N.C., Wilson I.A. (2020). Structural biology of influenza hemagglutinin: An amaranthine adventure. Viruses.

[39] Asthana S., Karna S.R., Shelby I.A. (2020). Amaranthine: Humanoid Robot Kinematics. Int. J. High Speed Electron. Syst..

[40] Snehalatha M., Ravikumar C., Sekar N., Jayakumar V.S., Hubert Joe I. (2008). FT-Raman, IR and UV-visible spectral investigations and ab initio computations of a nonlinear food dye amaranth. J. Raman Spectrosc..

[41] Shuai P., Guo Q., Liao L., Su K., Ding J., An N., Mei L., Woźny P., Runowski M. (2024). Na3GaF6:Ho3+,Yb3+@SiO2: A Novel Upconversion Sensor Material for Food Pigment Detection Application. Cryst. Growth Des..

[42] Moulya K.P., Manjunatha J.G., Aldossari S.A., Ataollahi N. (2025). Advanced graphene composite paste electrode for amaranth dye detection with tartrazine. Monatsh. Chem..

[43] Coy-Barrera E. (2020). Analysis of betalains (betacyanins and betaxanthins). Recent Advances in Natural Products Analysis.

[44] Jerz G., Gebers N., Szot D., Szaleniec M., Winterhalter P., Wybraniec S. (2014). Separation of amaranthine-type betacyanins by ion-pair high-speed countercurrent chromatography. J. Chromatogr. A.

[45] Kumorkiewicz-Jamro A., Górska R., Krok-Borkowicz M., Reczyńska-Kolman K., Mielczarek P., Popenda Ł., Spórna-Kucab A., Tekieli A., Pamuła E., Wybraniec S. (2023). Betalains isolated from underexploited wild plant *Atriplex hortensis* var. *rubra* L. exert antioxidant and cardioprotective activity against H9c2 cells. Food Chem..

[46] Schliemann W., Cai Y., Degenkolb T., Schmidt J., Corke H. (2001). Betalains of *Celosia argentea*. Phytochemistry.

[47] Schneider-Teixeira A., Molina-García A.D., Alvarez I., Dello Staffolo M., Deladino L. (2022). Application of betacyanins pigments from *Alternanthera brasiliana* as yogurt colorant. LWT-Food Sci. Technol..

[48] Escribano J., Cabanes J., Jiménez-Atiénzar M., Ibañez-Tremolada M., Gómez-Pando L.R., García-Carmona F., Gandía-Herrero F. (2017). Characterization of betalains, saponins and antioxidant power in differently colored quinoa (*Chenopodium quinoa*) varieties. Food Chem..

[49] Spórna-Kucab A., Hołda E., Wybraniec S. (2016). High-speed counter-current chromatography in separation of betacyanins from flowers of red *Gomphrena globosa* L. cultivars. J. Chromatogr. B Anal. Technol. Biomed. Life Sci..

[50] Sasaki N., Abe Y., Wada K., Koda T., Goda Y., Adachi T., Ozeki Y. (2005). Amaranthin in feather cockscombs is synthesized via glucuronylation at the cyclo-DOPA glucoside step in the betacyanin biosynthetic pathway. J. Plant Res..

[51] Stintzing F.C., Kammerer D., Schieber A., Adama H., Nacoulma O.G., Carle R. (2004). Betacyanins and Phenolic Compounds from *Amaranthus spinosus* L. and *Boerhavia erecta* L.. Z. Naturforsch.-Sect. C J. Biosci..

[52] Cai Y.-Z., Xing J., Sun M., Corke H. (2006). Rapid identification of betacyanins from *Amaranthus tricolor*, *Gomphrena globosa*, and *Hylocereus polyrhizus* by matrix-assisted laser desorption/ionization quadrupole ion trap time-of-flight mass spectrometry (MALDI-QIT-TOF MS). J. Agric. Food Chem..

[53] Spórna-Kucab A., Kumorkiewicz A., Szmyr N., Szneler E., Wybraniec S. (2019). Separation of betacyanins from flowers of *Amaranthus cruentus* L. in a polar solvent system by high-speed counter-current chromatography. J. Sep. Sci..

[54] Spórna-Kucab A., Jerz G., Kumorkiewicz-Jamro A., Tekieli A., Wybraniec S. (2021). High-speed countercurrent chromatography for isolation and enrichment of betacyanins from fresh and dried leaves of *Atriplex hortensis* L. var. “*Rubra*”. J. Sep. Sci..

[55] Schwarz S.J., Hildenbrand B.E., Von Elbe J.H. (1981). Comparison of Spectrophotometric and HPLC Methods to Quantify Betacyanins. J. Food Sci..

[56] Lystvan K., Kumorkiewicz A., Szneler E., Wybraniec S. (2018). Study on Betalains in *Celosia cristata* Linn. Callus Culture and Identification of New Malonylated Amaranthins. J. Agric. Food Chem..

[57] Hussain E.A., Sadiq Z., Zia-Ul-Haq M. (2018). Betalains: Biomolecular Aspects.

[58] Cai Y., Sun M., Corke H. (2001). Identification and distribution of simple and acylated betacyanins in the Amaranthaceae. J. Agric. Food Chem..

[59] Esquivel P. (2024). Chapter 6—Betalains. Handbook on Natural Pigments in Food and Beverages.

[60] Gandía-Herrero F., Escribano J., García-Carmona F. (2010). Structural implications on color, fluorescence, and antiradical activity in betalains. Planta.

[61] Kumorkiewicz-Jamro A., Górska R., Krok-Borkowicz M., Mielczarek P., Popenda Ł., Lystvan K., Pamuła E., Wybraniec S. (2023). Unveiling Alternative Oxidation Pathways and Antioxidant and Cardioprotective Potential of Amaranthin-Type Betacyanins from Spinach-like *Atriplex hortensis* var. ‘*Rubra*’. J. Agric. Food Chem..

[62] Cai Y., Sun M., Corke H. (2003). Antioxidant activity of betalains from plants of the Amaranthaceae. J. Agric. Food Chem..

[63] Roriz C.L., Xavier V., Heleno S.A., Pinela J., Dias M.I., Calhelha R.C., Morales P., Ferreira I.C.F.R., Barros L. (2021). Chemical and Bioactive Features of *Amaranthus caudatus* L. Flowers and Optimized Ultrasound-Assisted Extraction of Betalains. Foods.

[64] Sadowska-Bartosz I., Bartosz G. (2021). Biological Properties and Applications of Betalains. Molecules.

[65] Cai Y., Sun M., Corke H. (1998). Colorant Properties and Stability of *Amaranthus* Betacyanin Pigments. J. Agric. Food Chem..

[66] Sarker U., Oba S. (2019). Nutraceuticals, antioxidant pigments, and phytochemicals in the leaves of *Amaranthus spinosus* and *Amaranthus viridis* weedy species. Sci. Rep..

[67] Deladino L., Alvarez I., De Ancos B., Sánchez-Moreno C., Molina-García A.D., Schneider Teixeira A. (2017). Betalains and phenolic compounds of leaves and stems of *Alternanthera brasiliana* and *Alternanthera tenella*. Food Res. Int..

[68] Chen X.W., Shao L., Song L.Y., Chen Y.J., Peng C.L., Zhang Q. (2013). Amaranthine plays an important role in photoprotection for *Alternanthera sessilis* under photooxidative stress. Biotechnol. Biotechnol. Equip..

[69] Yap C.H., Mat Junit S., Abdul Aziz A., Kong K.W. (2019). Multiple extraction conditions to produce phytochemical- and antioxidant-rich *Alternanthera sessilis* (red) extracts that attenuate lipid accumulation in steatotic HepG2 cells. Food Biosci..

[70] Mohd Hazli U.H.A., Abdul-Aziz A., Mat-Junit S., Chee C.F., Kong K.W. (2019). Solid-liquid extraction of bioactive compounds with antioxidant potential from *Alternanthera sesillis* (red) and identification of the polyphenols using UHPLC-QqQ-MS/MS. Food Res. Int..

[71] Chang Y.J., Pong L.Y., Hassan S.S., Choo W.S. (2020). Antiviral activity of betacyanins from red pitahaya (*Hylocereus polyrhizus*) and red spinach (*Amaranthus dubius*) against dengue virus type 2 (GenBank accession no. MH488959). Access Microbiol..

[72] Yong Y.Y., Dykes G., Lee S.M., Choo W.S. (2017). Comparative Study of Betacyanin Profile and Antimicrobial Activity of Red Pitahaya (*Hylocereus polyrhizus*) and Red Spinach (*Amaranthus dubius*). Plant Foods Hum. Nutr..

[73] Niveyro S.L., Mortensen A.G., Fomsgaard I.S., Salvo A. (2013). Differences among five amaranth varieties (*Amaranthus* spp.) regarding secondary metabolites and foliar herbivory by chewing insects in the field. Arthropod. Plant. Interact..

[74] Gins E.M., Goryunova S.V., Motyleva S.M., Khasanova S.D., Gins V.K., Pivovarov V.F., Kulikov I.M., Baikov A.A., Gins M.S. (2024). Modulation of Low-Molecular-Weight Antioxidants in *Amaranthus tricolor* Leaves Exposed to Cold Stress During the Ripening Stage. SABRAO J. Breed. Genet..

[75] Kumorkiewicz-Jamro A., Pachulicz R.J., Fitter S., Górska R., Duggan J., Vandyke K., Pukala T.L., Wybraniec S., Zannettino A.C.W. (2025). *Atriplex hortensis* var. “*rubra*” extracts and purified amaranthin-type pigments reduce oxidative stress and inflammatory response in LPS-stimulated RAW264.7 cells. Food Chem..

[76] Das M., Saeid A., Hossain M.F., Jiang G.H., Eun J.B., Ahmed M. (2019). Influence of extraction parameters and stability of betacyanins extracted from red amaranth during storage. J. Food Sci. Technol..

[77] Cai Y.Z., Corke H. (2001). Effect of postharvest treatments on *Amaranthus* betacyanin degradation evaluated by visible/near-infrared spectroscopy. J. Food Sci..

[78] Khandaker L., Ali M.B., Oba S. (2009). Influence of cultivar and growth stage on pigments and processing factors on betacyanins in Red Amaranth (*Amaranthus tricolor* L.). Food Sci. Technol. Int..

[79] Sharma A., Mazumdar B., Keshav A. (2023). Valorization of unsalable *Amaranthus tricolour* leaves by microwave-assisted extraction of betacyanin and betaxanthin. Biomass Convers. Biorefin..

[80] Ahmed M., Ramachandraiah K., Jiang G., Eun J.B. (2020). Effects of Ultra-Sonication and Agitation on Bioactive Compounds and Structure of Amaranth Extract. Foods.

[81] Reis A., Kleinowski A.M., Klein F.R.S., de Souza R.T.T., do Amarante L., Braga E.J.B. (2017). Callus induction and betacyanin quantification by HPLC/MS-MS in *Alternanthera brasiliana* (L.) Kuntze. Hoehnea.

[82] Kleinowski A.M., Brandão I.R., Einhardt A.M., Ribeiro M.V., Peters J.A., Braga E.J.B. (2014). Pigment production and growth of Alternanthera plants cultured in vitro in the presence of tyrosine. Braz. Arch. Biol. Technol..

[83] Reis A., Kleinowski A.M., Klein F.R.S., Telles R.T., Do Amarante L., Braga E.J.B. (2015). Light quality on the in vitro growth and production of pigments in the genus Alternanthera. J. Crop Sci. Biotechnol..

[84] Bianco-Colomas J., Hugues M. (1990). Establishment and Characterization of a Betacyanin Producing Cell Line of *Amaranthus tricolor*: Inductive effects of Light and Cytokinin. J. Plant Physiol..

[85] Sang A., Roon T., Klanrit P., Klanrit P., Thanonkeo P., Apiraksakorn J., Thanonkeo S., Klanrit P. (2024). Establishment of Betalain-Producing Cell Line and Optimization of Pigment Production in Cell Suspension Cultures of *Celosia argentea* var. *plumosa*. Plants.

[86] Warhade M.I., Badere R.S. (2018). *Fusarium oxysporum* cell elicitor enhances betalain content in the cell suspension culture of Celosia cristata. Physiol. Mol. Biol. Plants.

[87] Berlin J., Sieg S., Strack D., Bokern M., Harms H. (1986). Production of betalains by suspension cultures of *Chenopodium rubrum* L.. Plant Cell Tissue Organ Cult..

[88] Beaumont M., Pandya Y., Knorr D. (1989). Chitosan immobilization and permeabilization of cultured *Apium graveolens*, *Chenopodium rubrum*, and *Daucus carota* cells. Food Biotechnol..

[89] Dörnenburg H., Knorr D. (1993). Cellular permeabilization of cultured plant tissues by high electric field pulses or ultra high pressure for the recovery of secondary metabolites. Food Biotechnol..

[90] Bokern M., Wray V., Strack D. (1991). Accumulation of phenolic conjugates and betacyanins, and changes in the activities of enzymes involved in feruloylglucose metabolism in cell-suspension cultures of *Chenopodium rubrum* L.. Planta.

[91] Bokern M., Heuer S., Strack D. (1992). Hydroxycinnamic Acid Transferases in the Biosynthesis of Acylated Betacyanins: Purification and Characterization from Cell Cultures of *Chenopodium rubrum* and Occurrence in Some Other Members of the Caryophyllales. Bot. Acta.

[92] Knorr D., Berlin J. (1987). Effects of Immobilization and Permeabilization Procedures on Growth of *Chenopodium rubrum* Cells and Amaranthin Concentration. J. Food Sci..

[93] Imamura T., Isozumi N., Higashimura Y., Miyazato A., Mizukoshi H., Ohki S., Mori M. (2019). Isolation of amaranthin synthetase from *Chenopodium quinoa* and construction of an amaranthin production system using suspension-cultured tobacco BY-2 cells. Plant Biotechnol. J..

[94] Glitz C., Dyekjær J.D., Solimando G.M.C., Avila Neto P.M., Rago D., Babaei M., Borodina I. (2025). Recombinant production of amaranthin and other betalain variants with yeast cell factories. Synth. Syst. Biotechnol..

[95] Thimmaraju R., Bhagyalakshmi N., Narayan M.S., Ravishankar G.A. (2003). Kinetics of pigment release from hairy root cultures of Beta vulgaris under the influence of pH, sonication, temperature and oxygen stress. Process Biochem..

[96] Pavlov A., Bley T. (2006). Betalains biosynthesis by *Beta vulgaris* L. hairy root culture in a temporary immersion cultivation system. Process Biochem..

[97] Milech C., do Amaral M.N., Auler P.A., Lucho S.R., Kleinowski A.M., da Maia L.C., Bianchi V.J., Braga E.J.B. (2023). Betalain accumulation and de novo transcriptome sequencing reveal the potential to increase bioactive compounds in *Alternanthera sessilis* elicited by methyl jasmonate. Acta Physiol. Plant..

[98] Zheng X., Liu S., Cheng C., Guo R., Chen Y., Xie L., Mao Y., Lin Y., Zhang Z., Lai Z. (2016). Cloning and expression analysis of betalain biosynthesis genes in *Amaranthus tricolor*. Biotechnol. Lett..

[99] Liu S., Zheng X., Pan J., Peng L., Cheng C., Wang X., Zhao C., Zhang Z., Lin Y., XuHan X. (2019). RNA-sequencing analysis reveals betalains metabolism in the leaf of *Amaranthus tricolor* L.. PLoS ONE.

[100] Bokern M., Strack D. (1988). Synthesis of hydroxycinnamic acid esters of betacyanins via 1-O-acylglucosides of hydroxycinnamic acids by protein preparations from cell suspension cultures of *Chenopodium rubrum* and petals of *Lampranthus sociorum*. Planta.

[101] Garay A.S., Towers G.H.N. (1966). Studies on the Biosynthesis of Amaranthin. Can. J. Bot..

[102] Sunnadeniya R., Bean A., Brown M., Akhavan N., Hatlestad G., Gonzalez A., Symonds V.V., Lloyd A. (2016). Tyrosine hydroxylation in betalain pigment biosynthesis is performed by cytochrome P450 enzymes in beets (*Beta vulgaris*). PLoS ONE.

[103] Xie H., Zeng J., Feng W., Gao W., Lai Z., Liu S. (2025). Differential Expression of Amaranth AtrDODA Gene Family Members in Betalain Synthesis and Functional Analysis of AtrDODA1-1 Promoter. Plants.

[104] Piattelli M., Giudici De Nicola M., Castrogiovanni V. (1969). Photocontrol of amaranthin synthesis in *Amaranthus tricolor*. Phytochemistry.

[105] Kochhar V.K., Kochhar S., Mohr H. (1981). An Analysis of the Action of Light on Betalain Synthesis in the Seedling of *Amaranthus caudatus*, var. *viridis*. Planta.

[106] Piattelli M., de Nicola M.G., Castrogiovanni V. (1971). The effect of kinetin on amaranthin synthesis in *Amaranthus tricolor* in darkness. Phytochemistry.

[107] Giudici De Nicola M., Piattelli M., Castrogiovanni V., Amico V. (1972). The effects of light and kinetin on amaranthin synthesis in relation to phytochrome. Phytochemistry.

[108] Stobart A.K., Pinfield N.J., Kinsman L.T. (1970). The effects of hormones and inhibitors on amaranthin synthesis in seedlings of *Amaranthus tricolor*. Planta.

[109] Kinsman L.T., Pinfield N.J., Stobart A.K. (1975). The hormonal control of amaranthin synthesis in *Amaranthus caudatus* seedlings. Planta.

[110] Giudici de Nicola M., Amico V., Piattelli M. (1975). Effects of light and kinetin on amaranthin synthesis induced by cAMP. Phytochemistry.

[111] Piattelli M., Giudici de Nicola M., Castrogiovanni V. (1970). The inhibition by actinomycin D and puromycin of light-stimulated amaranthin synthesis. Phytochemistry.

[112] French C.J., Pecket R.C., Smith H. (1973). Effect of light and exogenously applied precursors on amaranthin synthesis in *Amaranthus caudatus*. Phytochemistry.

[113] Sciuto S., Oriente G., Piattelli M., Impellizzeri G., Amico V. (1974). Biosynthesis of amaranthin in *Celosia plumosa*. Phytochemistry.

[114] Rajagopal R. (1980). Significance of cyclic nucleotides in amaranthin synthesis by *Amaranthus caudatus* seedlings. Ann. Bot..

[115] Köhler K.H., Dörfler M., Göring H. (1980). The influence of light on the cytokinin content of *Amaranthus* seedlings. Biol. Plant..

[116] Zhu C., Jin H., Guo Z., Li G., Xia K., Xu Z., Ng D., Gan L. (2016). Antagonistic effect of indole-3-acetic acid on kinetin-stimulated amaranthin accumulation in the cotyledons of *Amaranthus mangostanus* seedlings. J. Hortic. Sci. Biotechnol..

[117] Liu S., Wang X., Peng L. (2023). Comparative Transcriptomic Analysis of the Metabolism of Betalains and Flavonoids in Red Amaranth Hypocotyl under Blue Light and Dark Conditions. Molecules.

[118] Nana F.W., Hilou A., Millogo J.F., Nacoulma O.G. (2012). Phytochemical composition, antioxidant and xanthine oxidase inhibitory activities of *Amaranthus cruentus* L. and *Amaranthus hybridus* L. Extracts. Pharmaceuticals.

[119] Sarker U., Oba S., Alsanie W.F., Gaber A. (2022). Characterization of Phytochemicals, Nutrients, and Antiradical Potential in Slim Amaranth. Antioxidants.

[120] Fernando G.S.N., Sergeeva N.N., Vagkidis N., Chechik V., Marshall L.J., Boesch C. (2023). Differential Effects of Betacyanin and Betaxanthin Pigments on Oxidative Stress and Inflammatory Response in Murine Macrophages. Mol. Nutr. Food Res..

[121] Sarker U., Rabbani M.G., Oba S., Eldehna W.M., Al-Rashood S.T., Mostafa N.M., Eldahshan O.A. (2022). Phytonutrients, Colorant Pigments, Phytochemicals, and Antioxidant Potential of Orphan Leafy *Amaranthus* Species. Molecules.

[122] He Y., Hussain S.A., Dai W. (2025). Betanin Mitigates Inflammation and Ankle Joint Damage by Subduing the MAPK/NF-κB Pathway in Arthritis Triggered by Type II Collagen in Rats. Comb. Chem. High Throughput Screen..

[123] Martinez R.M., Longhi-Balbinot D.T., Zarpelon A.C., Staurengo-Ferrari L., Baracat M.M., Georgetti S.R., Sassonia R.C., Verri W.A., Casagrande R. (2015). Anti-inflammatory activity of betalain-rich dye of *Beta vulgaris*: Effect on edema, leukocyte recruitment, superoxide anion and cytokine production. Arch. Pharm. Res..

[124] Tyszka-Czochara M., Pasko P., Zagrodzki P., Gajdzik E., Wietecha-Posluszny R., Gorinstein S. (2016). Selenium Supplementation of Amaranth Sprouts Influences Betacyanin Content and Improves Anti-Inflammatory Properties via NFκB in Murine RAW 264.7 Macrophages. Biol. Trace Elem. Res..

[125] Yong Y.Y., Dykes G., Lee S.M., Choo W.S. (2019). Biofilm inhibiting activity of betacyanins from red pitahaya (*Hylocereus polyrhizus*) and red spinach (*Amaranthus dubius*) against *Staphylococcus aureus* and *Pseudomonas aeruginosa* biofilms. J. Appl. Microbiol..

[126] Yong Y.Y., Ong M.W.K., Dykes G., Choo W.S. (2021). Betacyanin-inhibited biofilm formation of co-culture of *Staphylococcus aureus* and *Pseudomonas aeruginosa* on different polymer surfaces. FEMS Microbiol. Lett..

[127] Lim C.M., Lal S.K., Isa N.M., Omar A.R., Choo W.S. (2024). Betacyanins from red pitahaya (*Hylocereus polyrhizus*) exhibit antiviral response against influenza A virus. Heliyon.

[128] Ahmadi H., Nayeri Z., Minuchehr Z., Sabouni F., Mohammadi M. (2020). Betanin purification from red beetroots and evaluation of its anti-oxidant and anti-inflammatory activity on LPS-activated microglial cells. PLoS ONE.

[129] Wang Y., Fernando G.S.N., Sergeeva N.N., Vagkidis N., Chechik V., Do T., Marshall L.J., Boesch C. (2022). Uptake and Immunomodulatory Properties of Betanin, Vulgaxanthin I and Indicaxanthin towards Caco-2 Intestinal Cells. Antioxidants.

[130] Xu X., Jiang Y., Yeo Q.X., Zhou W. (2024). Purification and characterization of betacyanin monomers from *Hylocereus polyrhizus* peel: A comparative study of their antioxidant and antidiabetic activities with mechanistic insights. Food Chem..

[131] Cai Y., Corke H. (1999). *Amaranthus* betacyanin pigments applied in model food systems. J. Food Sci..

[132] Khan M.I. (1980). Gibberellic acid bioassay based on the inhibition of anthocyanin production in tomato seedlings. Biol. Plant..

[133] Yu J., Deng S., Huang H., Mo J., Xu Z.F., Wang Y. (2023). Exploring the Potential Applications of the Noninvasive Reporter Gene RUBY in Plant Genetic Transformation. Forests.

[134] Azeredo H.M.C. (2009). Betalains: Properties, sources, applications, and stability—A review. Int. J. Food Sci. Technol..

[135] Khan M.I. (2016). Plant Betalains: Safety, Antioxidant Activity, Clinical Efficacy, and Bioavailability. Compr. Rev. Food Sci. Food Saf..

[136] Klanrit P., Thanonkeo S., Klanrit P., Klanrit P., Mueangnak K., Thanonkeo P. (2025). *Celosia argentea*: Towards a Sustainable Betalain Source—A Critical Review and Future Prospects. Plants.

